# MFN2 mutations in Charcot–Marie–Tooth disease alter mitochondria-associated ER membrane function but do not impair bioenergetics

**DOI:** 10.1093/hmg/ddz008

**Published:** 2019-01-11

**Authors:** Delfina Larrea, Marta Pera, Adriano Gonnelli, Rubén Quintana–Cabrera, H Orhan Akman, Cristina Guardia-Laguarta, Kevin R Velasco, Estela Area-Gomez, Federica Dal Bello, Diego De Stefani, Rita Horvath, Michael E Shy, Eric A Schon, Marta Giacomello

**Affiliations:** 1 Department of Neurology, Columbia University Medical Center, New York, NY, USA; 2 Department of Biology, University of Padova 35131, Italy; 3 Department of Pathology and Cell Biology, Columbia University Medical Center, New York, NY, USA; 4 Department of Biomedical Sciences, University of Padova, Italy; 5 Institute of Genetic Medicine, Newcastle University, Newcastle upon Tyne, UK; 6 Department of Neurology, University of Iowa, Iowa City, IA, USA; 7 Department of Genetics and Development, Columbia University Medical Center, New York, NY, USA

## Abstract

Charcot–Marie–Tooth disease (CMT) type 2A is a form of peripheral neuropathy, due almost exclusively to dominant mutations in the nuclear gene encoding the mitochondrial protein mitofusin-2 (*MFN2*). However, there is no understanding of the relationship of clinical phenotype to genotype. MFN2 has two functions: it promotes inter-mitochondrial fusion and mediates endoplasmic reticulum (ER)–mitochondrial tethering at mitochondria-associated ER membranes (MAM). MAM regulates a number of key cellular functions, including lipid and calcium homeostasis, and mitochondrial behavior. To date, no studies have been performed to address whether mutations in *MFN2* in CMT2A patient cells affect MAM function, which might provide insight into pathogenesis. Using fibroblasts from three CMT2A^MFN2^ patients with different mutations in *MFN2*, we found that some, but not all, examined aspects of ER–mitochondrial connectivity and of MAM function were indeed altered, and correlated with disease severity. Notably, however, respiratory chain function in those cells was unimpaired. Our results suggest that CMT2A^MFN2^ is a MAM-related disorder but is not a respiratory chain-deficiency disease. The alterations in MAM function described here could also provide insight into the pathogenesis of other forms of CMT.

## Introduction

Charcot–Marie–Tooth disease Type 2A (CMT2A; OMIM #609260) is a neurological disorder that presents as a peripheral neuropathy (1) but involves the central nervous system (2). This autosomal-dominant disorder is due to heterozygous mutations in the gene encoding mitofusin-2 (*MFN2*) (3) but occasionally *MFN2* mutations are recessive (4) or semi-dominant (5). Clinically, patients exhibit progressive sensory loss in the extremities and *pes cavus*, among other features (6,7), with the disease presenting as early- or late-onset forms (8). Studies in relevant tissues, such as sural nerve, have shown consistent phenotypes in which chronic axonal atrophy and subsequent regeneration are often seen (9). Approximately 100 pathogenic mutations in *MFN2* have been described (10), but there is no understanding of the relationship of clinical phenotype to genotype.

MFN2 and its paralog MFN1 localize to the outer mitochondrial membrane to promote inter-mitochondrial fusion (11). Both mitofusins protrude into the cytoplasm and establish homotypic and heterotypic interactions (12). Importantly, MFN2, but not MFN1, also localizes to a subdomain of the endoplasmic reticulum (ER) called mitochondria-associated ER membranes (MAM) and plays a key role in tethering the two organelles (13,14). MFN2 contains Ras-binding and GTPase domains, two transmembrane domains and two heptad repeat (HR) domains (11) presumably important for inter-mitochondrial fusion (15). Recently, a re-evaluation of MFN2 topology showed that it has a single membrane-spanning domain where redox-mediated disulfide modifications could drive MFN2 oligomerization and mitochondrial fusion (16,17).

Morphological studies of nervous tissue and fibroblasts from CMT2A patients with mutations in *MFN2* (henceforth denoted CMT2A^MFN2^) displayed altered mitochondrial morphology, including swelling, degeneration and altered distribution of mitochondria, with an increase in the number of mitochondria in paranodal extensions of axons, in myelin loops and in unmyelinated axons (9,18). Interestingly, however, no fragmentation of the organelles was observed in those studies, contrary to what has been observed in cellular models in which mutated *MFN2* was overexpressed (19,20). Mitochondrial alterations are also apparent in Schwann cells of myelinated fibers, with the occurrence of needle-like calcium precipitates and electron-dense lysosome-like structures (21). However, studies of mitochondria in patient fibroblasts have reported extremely variable and often contradictory findings, with little agreement regarding whether or not there are alterations in MFN2 protein levels (18,22), in respiratory chain capacity and oxidative phosphorylation (18,22,23), in mitochondrial membrane potential (23) or in mtDNA content (18,22). In addition, mitochondrial respiration and MFN2 protein stability were not affected in HEK-293T cells overexpressing either wild-type (WT) or pathogenic CMT2A-mutant *MFN2* (19).

Studies in transgenic mice have been equally difficult to interpret. Mice overexpressing a pathogenic mutation in *MFN2* (R94Q) associated with CMT2A^MFN2^ in neurons showed altered mitochondrial distribution and respiratory chain deficiencies (24). In mouse dorsal root ganglia overexpressing WT MFN2 or CMT2A^MFN2^ proteins, mitochondrial transport, distribution and morphology along the axons were altered significantly, but ATP levels and mitochondrial membrane potential were unaffected (25–27).

Finally, motor neurons derived from induced pluripotent cells obtained from fibroblasts from a CMT2A^MFN2^ patient could recapitulate some, but not all, of the disease-related features, including abnormal mitochondrial trafficking and cytoskeletal distribution, but mitochondrial morphology was unchanged and many examined phenotypes were relatively mild (28). It is possible that the heterogeneous findings found in the various CMT2A models are related to the degree of protein expression and the ratio between WT and mutant MFN2 species.

As noted above, MFN2 mediates the tethering between mitochondria and the ER at MAM (13,14). MAM regulates a number of key cellular functions, among them lipid and calcium homeostasis, mitochondrial dynamics and bioenergetics (29,30). More recently it has been shown that MFN2 tethering can be regulated by post-transcriptional modifications upon non-degradative ubiquitination of MFN2 by the E3 ligase MARCH5 (31). Notably, loss of MARCH5 reduces ER–mitochondrial tethering and Ca^2+^ transfer without affecting mitochondrial or ER morphology (31). Conversely, in cells lacking MFN2, inter-oraganellar connectivity and lipid homeostasis are severely affected (32). It is not known how mutations in *MFN2* affect this communication and whether those potential changes are related to disease progression. In particular, no examination of MAM function has been performed, either in cellular models or in patient cells. To address this gap in our understanding, we selected fibroblasts from three patients who not only harbored different mutations in *MFN2*, but also presented with a variety of clinical phenotypes, ranging from mild to severe, and studied them in some detail from both the MAM and mitochondrial point of view. We felt that this integrated approach could help explain some of the features associated with the pathogenesis of CMT2A disease.

## Results

### Expression of *MFN2* mRNA and protein in CMT2A^MFN2^ fibroblasts

CMT2A is a dominant neuropathy in which patients are heterozygous for *MFN2* mutations, but it is not known whether the CMT2A phenotype results from *MFN2* haploinsufficiency is a dominant negative effect on the WT allele or is semi-dominant. To gain more insight into the possible relationship between the WT and mutant alleles, we analyzed fibroblasts from three CMT2A patients harboring pathogenic dominant mutations in *MFN2*: R364W (patient 1; P1), M376V (P2) and W740S (P3), and age and sex matched controls (Table 1). The location of the mutation in severely affected patient 1 (at aa-364; see Materials and Methods) is in the area between the GTPase domain and HR1; the mutation in moderately affected patient 2 (at aa-376) is located within HR1, in the region required for the fusion-competent ‘open’ conformation of MFN2 (20); and the mutation in mildly-affected patient 3 (at aa-740) is in HR2 (33).

**Table 1:** Patient and control fibroblasts

**Fibroblast**	**Code #**	**Age**	**Sex**	**Mutation**	**Source**
Patient P1		32	F	R364W	M. Shy
Patient P2		60	M	M376 V	R. Horvath
Patient P3		43	F	W740S	M. Shy
Control C1	F253-12N				R. Horvath
Control C2	F011-11N	45	F		R. Horvath
Control C3	AG06858	47	M		Coriell
Control C4	AG02261	61	M		Coriell
Control C5	AG02222	49	M		Coriell
Control C6	CUMC01	30	F		CUMC

**Figure 1:**
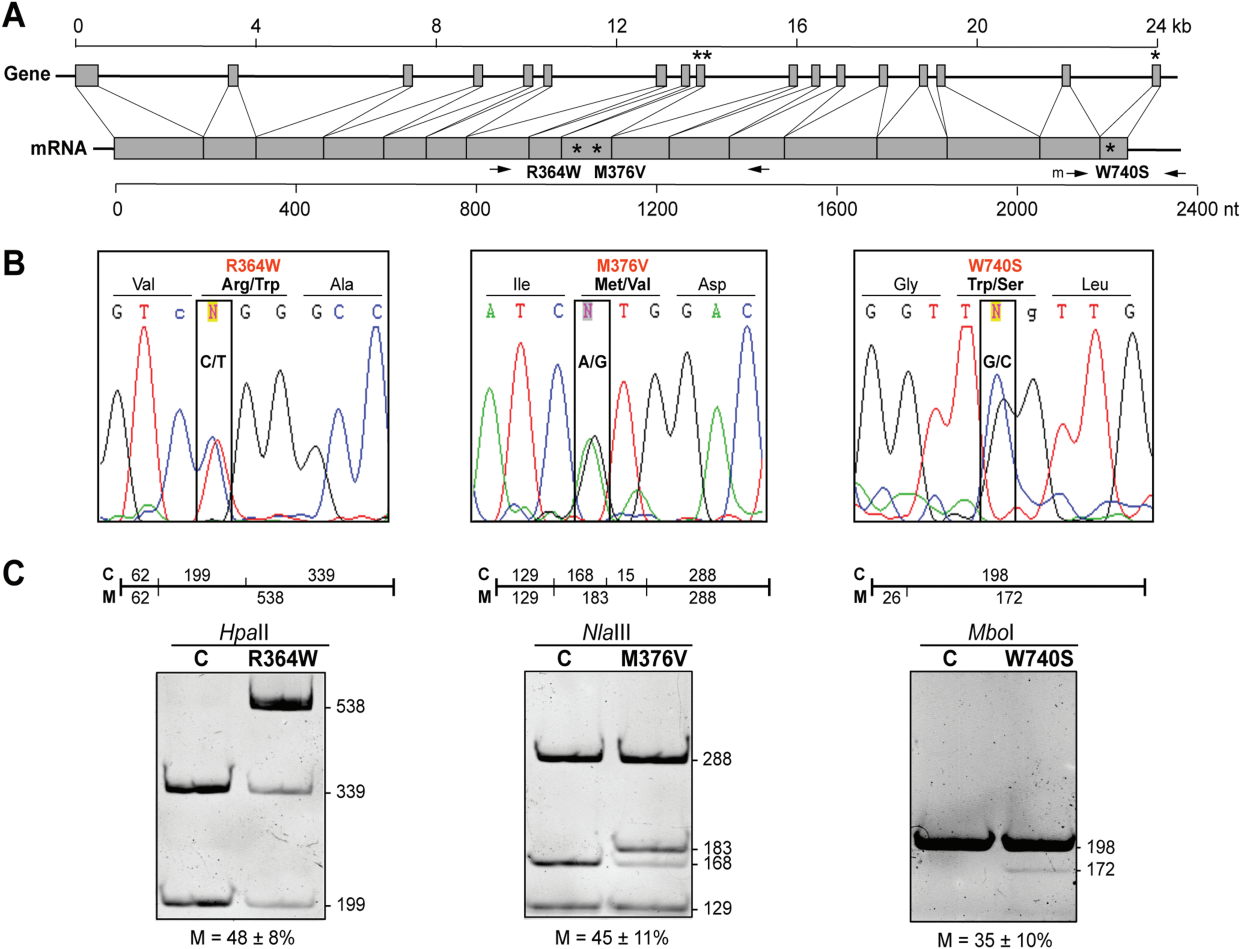
Genetic analysis of CMT2A^MFN2^ patients. **(A)** Maps of the human MFN2 gene (exons, gray boxes; size, in kb, above the gene) and the processed mRNA (size, in nt, below the mRNA). Asterisks denote locations of the mutations in the three CMT2A^MFN2^ patients. **(B)** Pherograms of the region in the mRNA encompassing the indicated mutations (amplified by RT-PCR using the primers denoted by the arrows in A). Note the approximately equal expression of the mRNAs derived from the WT and mutant alleles (boxed). **(C)** RFLP analysis of the region of *MFN2* mRNA amplified by RT-PCR and cleaved with the indicated restriction enzymes, and whose cleavage pattern in the gel corresponds to the cleavage map shown above each gel; *n* = 3, 4 and 3 for R364W, M376V and W740S, respectively. For R364W and M376V, the % mutation was not significantly different from the expectation of 50%. For W740S, for which no natural restriction site was present, and for which the cDNA was amplified using a mismatched primer (Supplementary Material, Fig. **S2**), there was borderline significance (*P* = 0.049). C, control. M, mutant. Arrows denote various PCR primers; m, mismatched primer (A).

**Figure 2:**
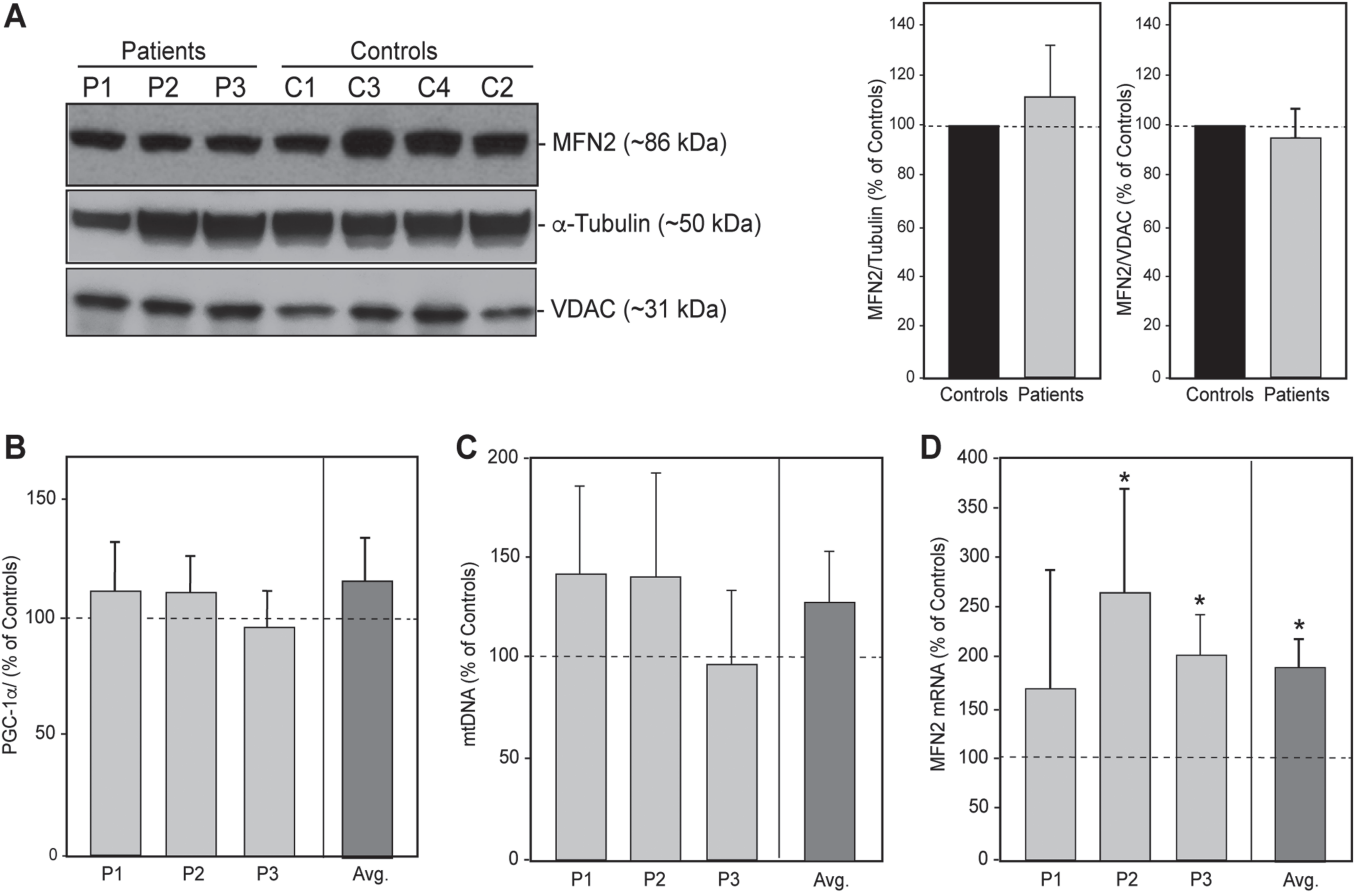
Analysis of MFN2 expression in CMT2A^MFN2^ patient fibroblasts. **(A)** Representative western blots of total protein from fibroblasts from four controls and the three patients (identified in Table 1), probed for the indicated markers, relative to the expression of α-tubulin and VDAC (predicted molecular masses in parentheses); 20 μg loaded in each lane. Quantitation at right. **(B)** Expression of total PGC-1α mRNA in patients relative the control average (*n* = 6; set at 100%). *n* = 3. **(C)** Quantitation of mtDNA relative to control average (*n* = 5; set at 100%). **(D)** Expression of total *MFN2* mRNA relative to that in the control average (*n* = 6; set at 100%). Avg., average of all 3 patients; ^*^P < 0.05.

The heterozygous nature of the mutations was confirmed by DNA sequencing (Fig. 1A and data not shown). We then quantified the steady-state levels of MFN2 protein in total homogenates of controls and the three CMT2A^MFN2^ patients by western blot. The total amount of MFN2 protein was unchanged in patients compared to that in controls (Fig. 2A), nor was that of its paralog, MFN1 (Supplementary Material, Fig. S1). Importantly, mitochondrial mass, as measured by the amount of VDAC protein (Fig. 2A) and the mRNA level of the mitochondrial master regulator PGC-1α (Fig. 2B), or of mtDNA (Fig. 2C), were unaltered in the patients. In contrast, average steady-state levels of *MFN2* mRNA were ~2-fold higher in the patients than in controls (Fig. 2D).

The ~2-fold increase in *MFN2* mRNA was unexpected and implied that there might be altered expression of *MFN2* mRNA from the WT and mutant alleles. We therefore sequenced *MFN2* cDNA derived from reverse transcription polymerase chain reaction (RT-PCR) of total mRNA, using *MFN2*-specific primers (Fig. 1A and B); in all three patient fibroblasts, we detected expression from both alleles, in approximately equal amounts (Fig. 1B). In order to determine whether there was differential transcription from the WT and mutant alleles, we performed restriction fragment length polymorphism (RFLP) analysis of these RT-PCR-amplified cDNAs, using restriction enzymes that would enable us to distinguish, and quantitate, the relative expression from the two alleles (Fig. 1C and Supplementary Material, Fig. S2). When quantitating the amount of WT and mutant cDNA derived from the respective alleles, we were careful to make the proper corrections, both for fragment size and for formation of heteroduplexes that were resistant to digestion (see Materials and Methods). For the R364W (loss of a *Hpa*II site) and M376V (loss of an *Nla*III site) mutations, there were essentially equal steady-state levels of *MFN2* mRNA derived from the WT and mutant alleles (48 ± 8% and 45 ± 11% mutant, respectively) (Fig. 1C). For the W740S mutation [gain of a *Mbo*I site introduced by the mismatched PCR primer (Supplementary Material, Fig. S2)], however, there was a reduction in the steady-state level of the mutant, from an expected level of 50% to 35 ± 10%, but this value was at the borderline of significance (*n* = 3, *P* = 0.049) (Fig. 1C). Taken together, we believe that the mutant *MFN2* alleles were expressed at essentially the same steady-state level as the WT alleles.

### Mitochondrial morphology in CMT2A^MFN2^ fibroblasts

**Figure 3:**
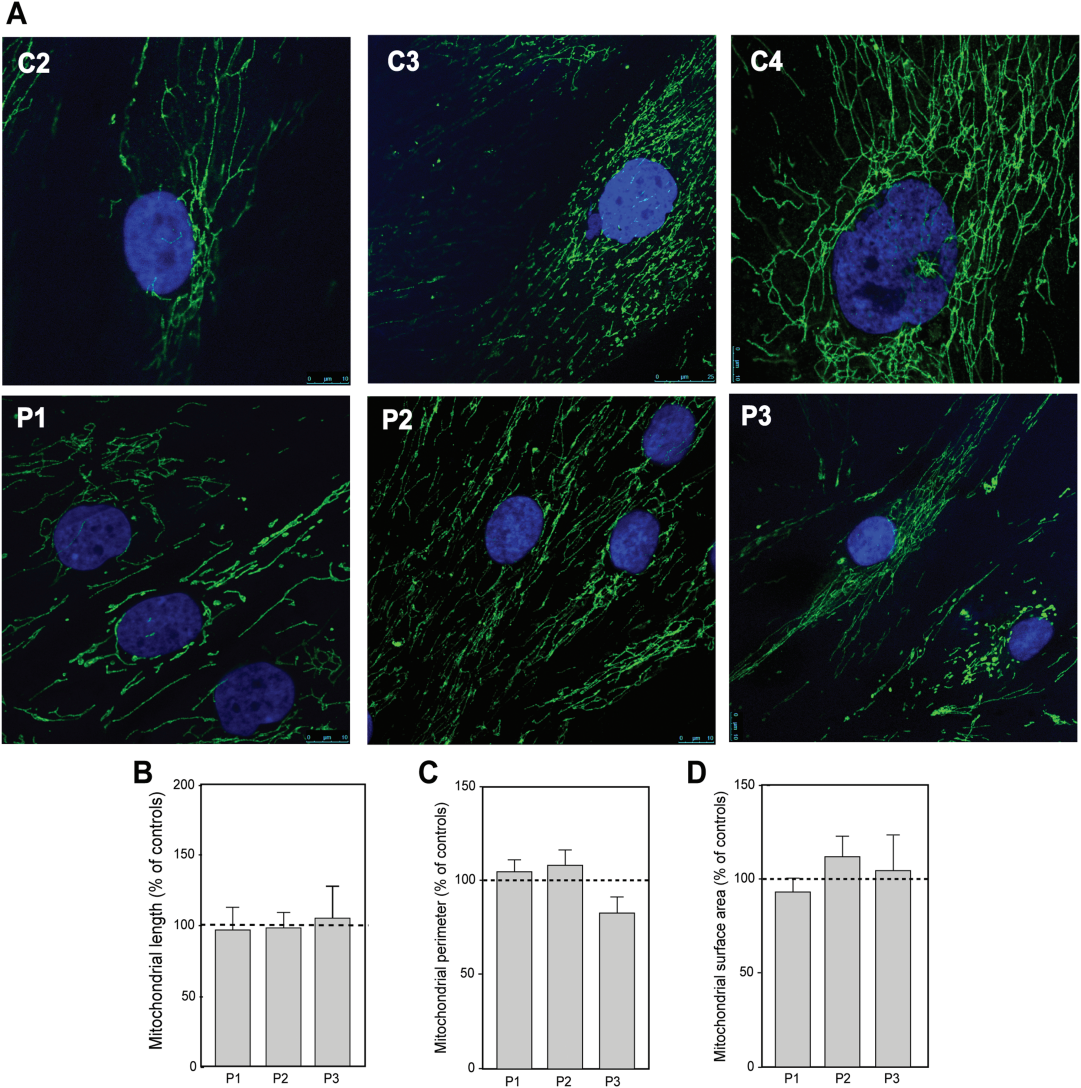
Morphology of mitochondria in CMT2A^MFN2^ patient fibroblasts. **(A)** Representative confocal microscopy images of the mitochondrial network, as visualized by anti-TOM20 (shown here in green); 4′,6-diamidino-2-phenylindole (DAPI)-stained nuclei are in blue. **(B)** Mitochondrial length, measured by confocal microscopy. **(C)** Mitochondrial perimeter, measured by EM. **(D)** Mitochondrial surface area, measured by EM.

Mouse embryonic fibroblasts (MEFs) lacking *Mfn2* have fragmented mitochondria (34), whereas expression of the mouse homologs of various pathogenic human *MFN2* mutations (among them the W740S mutation studied here) in both WT-MEFs and in double-knockout MEFs lacking *Mfn1* and *Mfn2* display a variety of morphological phenotypes (19), ranging from highly tubular to highly aggregated (including W740S, when overexpressed). We therefore studied mitochondrial morphology in the three patient fibroblasts by confocal microscopy. The mitochondrial network in the patients’ cells looked similar to that in the controls (Fig. 3A), as has been observed by others (18), with no alteration in the length of the organelles (Fig. 3B). In addition, using electron microscopy (EM), which allows for the direct observation of organelle morphology, we found that there was no significant difference in mitochondrial perimeter or surface area in patients versus controls (Fig. 3C and D).

### ER–mitochondrial contacts in CMT2A^MFN2^ fibroblasts

**Figure 4:**
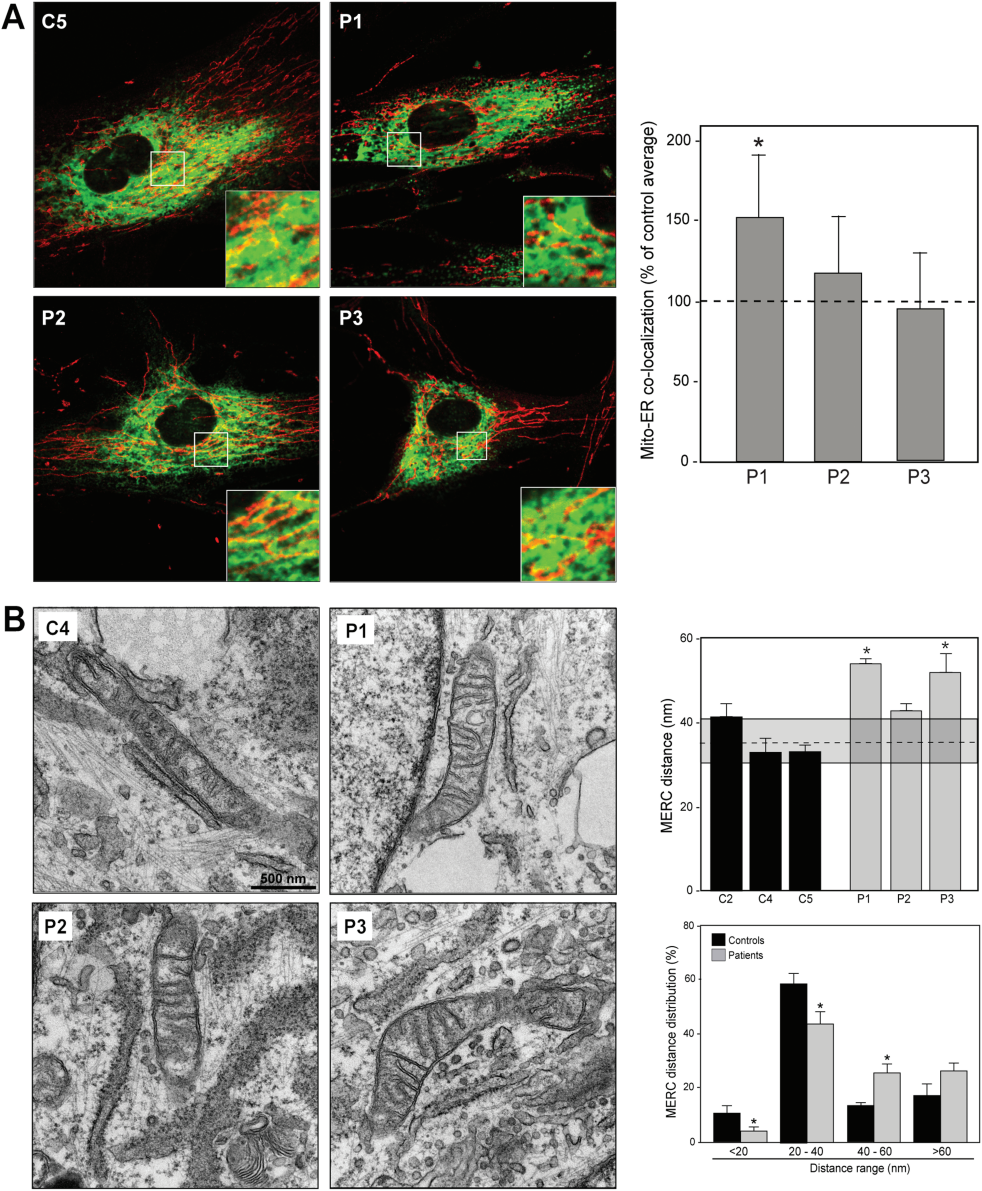
ER–mitochondrial connectivity in CMT2A^MFN2^ fibroblasts. **(A)** Representative confocal microscopy of ER (anti-PDI; green) and mitochondria (anti-TOM20; red). Quantification at right. **(B)** Representative EM pictures of ER and mitochondria in the indicated cells. Quantitation of mitochondria–ER contacts (MERC), a measurement of inter-organellar distance. The dotted line within the shaded box denotes the control average ± SD (top right), and histograms of the distribution of ER–mitochondrial distances in the averages of the controls and the patients (bottom right). *n* = 3; ^*^P < 0.05. Other parameters of ER–mitochondrial apposition are shown in Supplementary Material, Figure S3.

Aside from its role in inter-mitochondrial fusion, MFN2 participates in ER–mitochondrial tethering and communication (13). In order to examine this aspect of MFN2 function, we assessed ER–mitochondrial apposition, both by confocal and EM (Fig. 4). In confocal microscopy, we measured ER–mitochondrial apposition indirectly, defined as the visual overlap between markers of ER (in green) and mitochondria (in red) (i.e. yellow). By this criterion (which admittedly is a relatively low-resolution technique that allows one to detect inter-organellar distances of only ~200 nm; 35), the overlap of fluorescent signals was increased in patient 1 versus controls but was unchanged in patients 2 and 3 (Fig. 4A). It was unclear whether the increased co-localization in patient 1 was due to a greater number of contacts, an increase in the length of contacts or some combination of the two. Using EM, measurement of inter-organellar distance at regions of apposition [i.e. mitochondria–ER contact (MERC) distance; 36)] showed a significantly greater distance between the ER and mitochondria in patients 1 and 3 (Fig. 4B). We also found an increase in both the length of the inter-organellar contacts (MERC length; 36) and in the ER–mitochondria contact coefficient (ERMICC), which takes into account the ER–mitochondrial distance, the length of the contact and the mitochondrial perimeter (14), in patient 1, but not in patients 2 or 3 (Supplementary Material, Fig. S3A and B), consistent with the confocal data.

We also ordered the MERC distances into four size categories (12–20 nm, 20–40 nm, 40–60 nm and >60 nm) and found that, in broad view, there were fewer contacts with a cleft width <40 nm and higher number of contacts >40 nm in all three patients as compared to controls (Fig. 4B and Supplementary Material, Fig. S3C), consistent with the greater inter-organellar distances.

Taken together, these data show that these patient cells are characterized by a lower degree of physical association between mitochondria and ER as compared to controls.

### MAM function in CMT2A^MFN2^ fibroblasts

The structural analysis of MAM obtained by microscopic approaches does not take into account the dynamic nature of ER–mitochondrial communication. As a functional readout of MAM dynamics, we focused on three hallmarks of MAM function that would provide such insight, namely phospholipid transport/synthesis (37,38), cholesteryl ester (CE) synthesis (32,39) and calcium trafficking (40,41).

#### Phospholipid synthesis and trafficking

**Figure 5:**
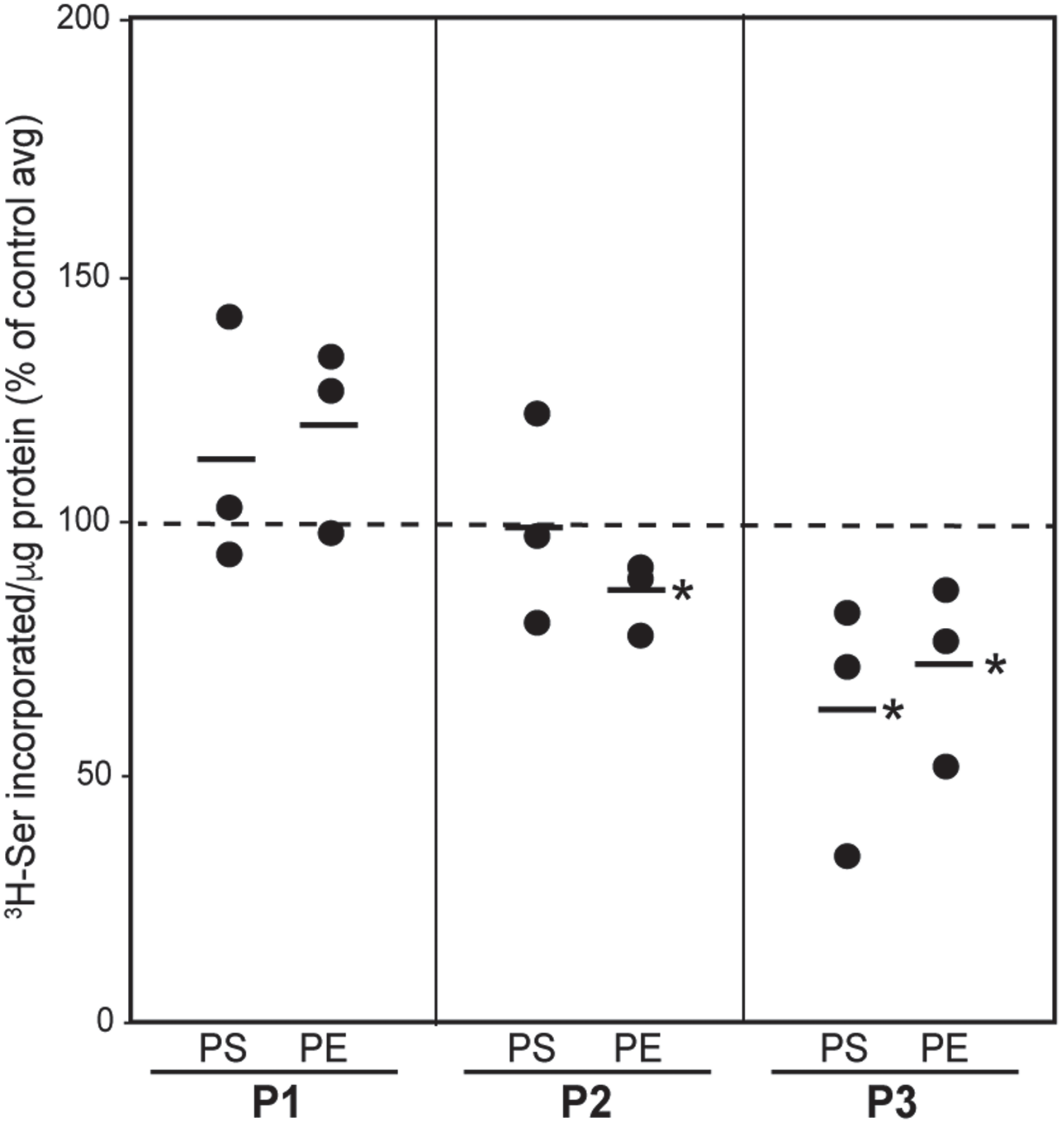
Phospholipid synthesis in CMT2A^MFN2^ fibroblasts. Incorporation after 4 h of ^3^H-Ser into ^3^H-PtdSer (PS) and ^3^H-PtdEtn (PE) in the indicated patient fibroblasts, relative to that of the average in the controls (set at 100%; based on the data shown in Supplementary Material, Fig. S4). Each data point represents the value in one experiment (i.e. one or more patients compared to controls, all analyzed on the same day). Bars indicate average values (*n* = 3 experiments); ^*^P < 0.05 versus control average.

Both mitochondria and ER play key roles in the synthesis of phosphatidylserine (PtdSer) and phosphatidylethanolamine (PtdEtn). PtdSer is synthesized in the MAM; it then translocates to mitochondria, where it is converted to PtdEtn; PtdEtn then translocates back to the MAM (37). Therefore, to test directly the effect of *MFN2* mutations on phospholipid synthesis mediated by MAM, we incubated patient and control fibroblasts in medium containing ^3^H-serine and measured the incorporation of the label into newly-synthesized ^3^H-PtdSer and ^3^H-PtdEtn after 2 and 4 h. In all cells, the degree of incorporation increased over time, as expected (Supplementary Material, Fig. S4), but there was no consistent pattern of serine incorporation among the patients. Specifically, patient 3 had significantly reduced levels of ^3^H-PtdSer and ^3^H-PtdEtn compared to controls, which suggests a reduced crosstalk between ER and mitochondria in this patient, whereas the other two patients showed little or no reduction (Fig. 5).

#### CE synthesis

**Figure 6:**
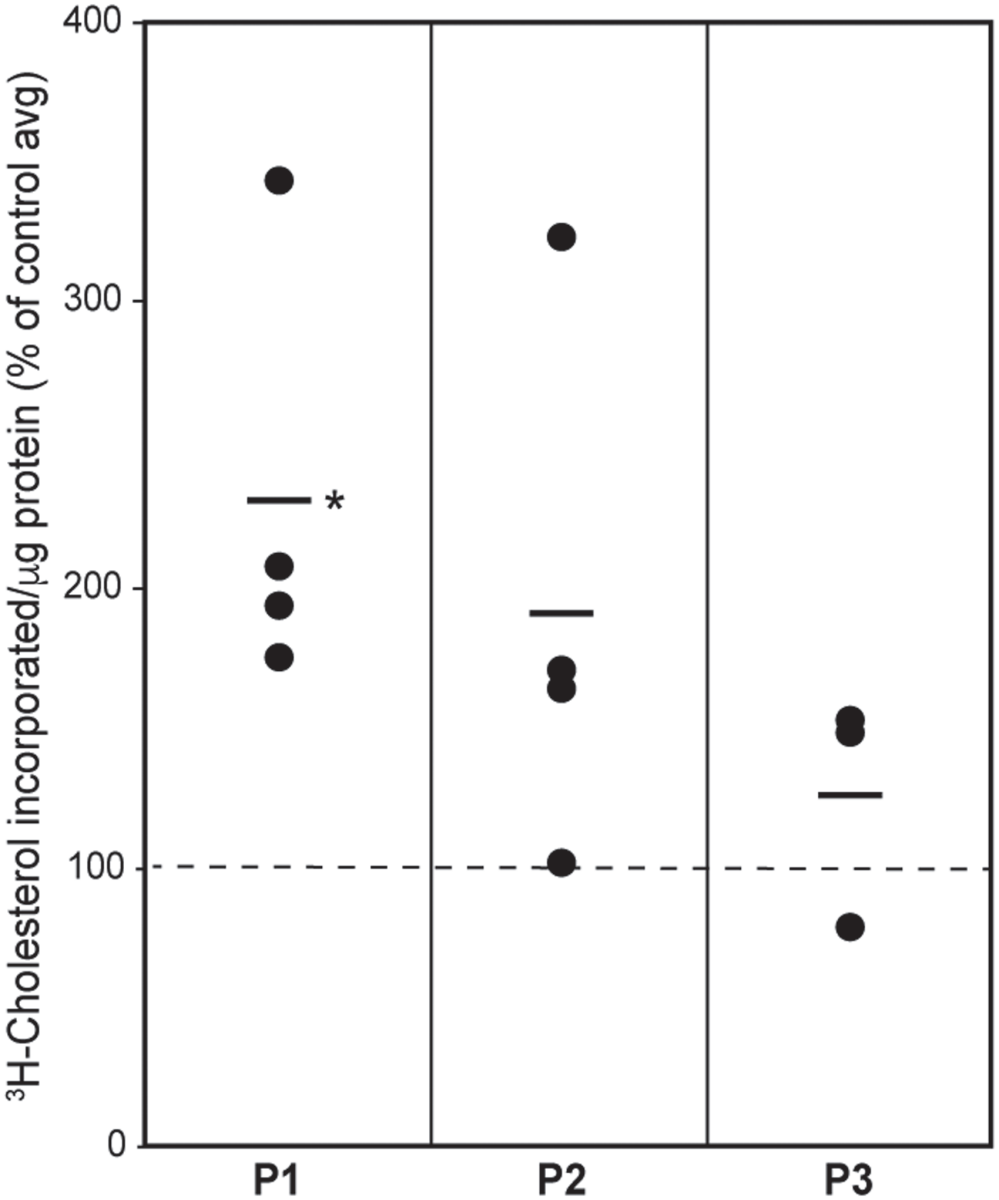
CE synthesis in CMT2A^MFN2^ fibroblasts. Incorporation of ^3^H-cholesterol into ^3^H-CE after 6 h in the indicated patient fibroblasts, relative to that of the average in the controls (set at 100%; based on the data shown in Supplementary Material, Fig. S5). Each data point represents the value in one experiment. Bars indicate average values (*n* = 3 experiments); ^*^P < 0.05 versus control average.

Cholesterol is converted to CE by acyl-CoA:cholesterol acyltransferase 1 (ACAT1; gene *SOAT1*), a MAM-localized enzyme (39); excess CE are deposited in lipid droplets (LDs) in the cytosol (32,42). We therefore incubated patient and control cells with ^3^H-cholesterol and measured its conversion into ^3^H-CE by ACAT1 after 6 h of incubation (Supplementary Material, Fig. S5). Contrary to what we saw in the phospholipid transfer assay, we found a significant increase in CE synthesis in patient 1 and a marginal increase in patient 2, whereas the CE level in patient 3 was essentially unchanged (Fig. 6).

**Figure 7:**
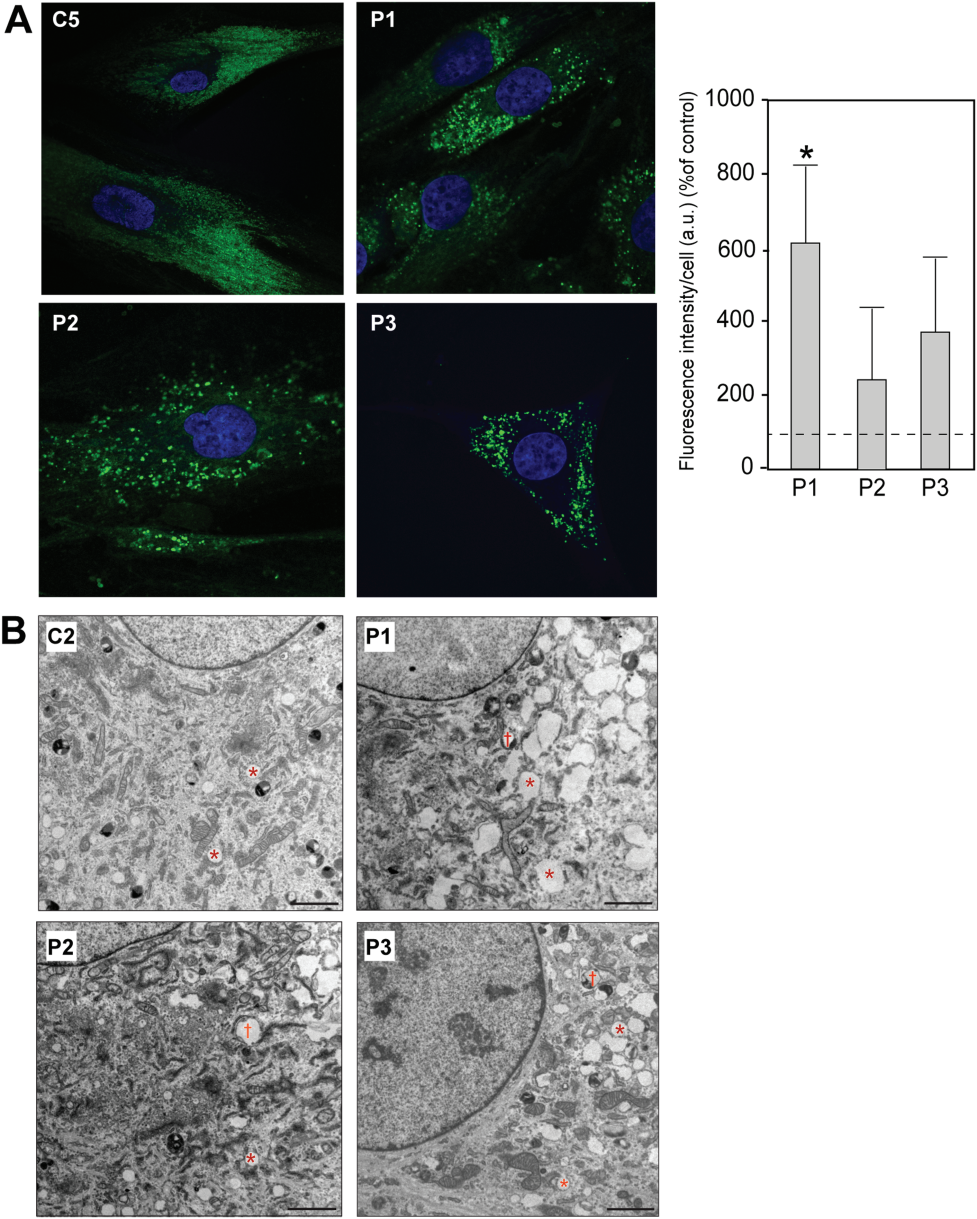
Accumulation of LDs in CMT2A^MFN2^ fibroblasts. **(A)** Representative examples of staining of the indicated cells with LipidTox Green™. Quantitation of LD accumulation in control average (dotted line) and patient cells at right. a.u., arbitrary units; ^*^P < 0.05. **(B)** EMs of the indicated cells, showing organelles that appear to be LDs (asterisks) as well as what appear to be autophagosomes containing LDs (daggers), indicative of lipophagy. Scale bars = 2 μm.

We also looked for the presence of LDs after staining cells with HCS LipidTox Green™. We detected the most LDs in fibroblasts from patient 1, and although not statistically significant, we observed a clear trend toward increased LDs in the other two patients (Fig. 7A). These data were consistent with the CE data, in which patient 1 had the greatest CE synthesis (Fig. 6). In addition, we observed numerous structures that appeared to be LDs in EM images of patient fibroblasts compared to control (asterisks in Fig. 7B), plus partially electron-dense objects that appeared to be autophagosomes containing what appeared to be LDs (daggers in Fig. 7B), perhaps indicative of lipophagy, similar to what has been reported by others (21). We note that LDs also accumulate in another peripheral neuropathy, hereditary sensory neuropathy type 1 (43,44), which is due to dominant mutations in serine palmitoyltransferase 1, a protein involved in sphingolipid synthesis (see also 45,46).

#### Calcium homeostasis

MFN2 modulates intracellular Ca^2+^ handling, as the ER Ca^2+^ content in *Mfn2*-knockout cells is higher than in WT cells (13). The tethering between the ER and mitochondria also impinges on Ca^2+^ homeostasis by controlling the transfer of this cation between the two organelles (13,41).

**Figure 8:**
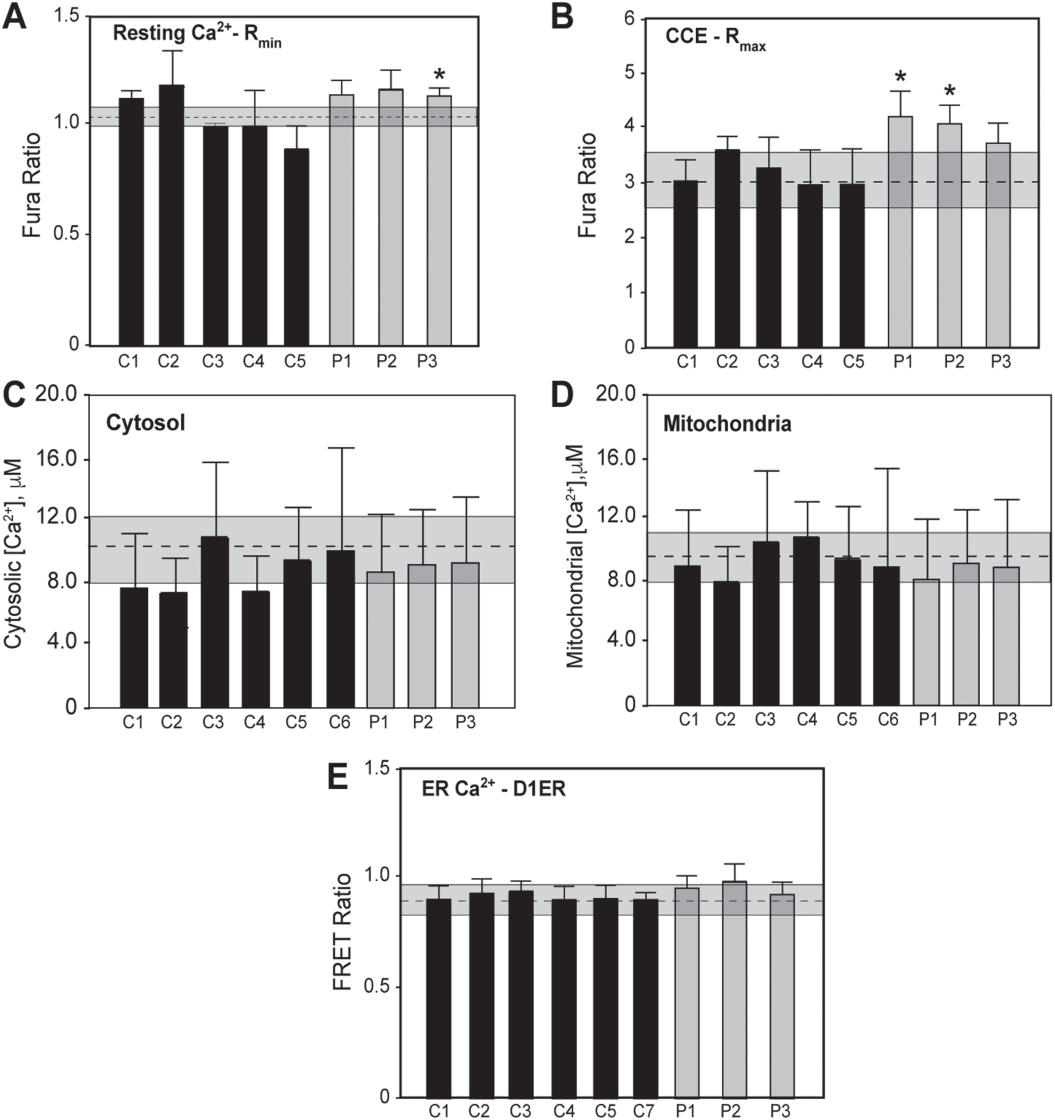
Calcium homeostasis in CMT2A^MFN2^ fibroblasts. **(A)** Measurement of cytosolic Ca^2+^ in the absence of added Ca^2+^ or any stimuli, using cells loaded with Fura-2-AM; changes in [Ca^2+^] are represented by the 340/380 ratio (R_min_). **(B)** Measurement of cytosolic Ca^2+^ upon treatment with 1 μM thapsigargin followed by the addition of 0.1 mM Ca^2+^ (i.e. to measure CCE), using cells loaded with Fura-2-AM; [Ca^2+^] measured as 340/380 ratio (R_max_). **(C and D)** Measurement of cytosolic Ca^2+^ and mitochondrial Ca^2+^ uptake, using cytosolic- and mitochondrial-targeted aequorins, respectively, after depletion of intracellular Ca^2+^ stores with thapsigargin. **(E)** Measurement of ER calcium content. Cells were transfected transiently with D1ER, a cameleon targeted to the ER, and the FRET ratio was measured in the absence of any stimuli. All results represent mean ± SD of at least three independent experiments. ^*^Significant versus the average of all the controls (*P* < 0.05). In each panel, the dotted line within the shaded box denotes the control average ± SD.

We hypothesized that pathogenic mutations in *MFN2* could affect MAM-mediated Ca^2+^ trafficking. We first loaded cells with Fura-2-AM and measured cytosolic Ca^2+^ in the absence of any stimuli. In this baseline condition there were no significant differences in Ca^2+^ content between patients and controls (Fig. 8A). Similarly, using D1ER, a calcium-sensitive (cameleon) probe targeted to the lumen of the ER (47), we saw no differences in ER Ca^2+^ content in unstimulated cells from all three patients (Fig. 8E). In order to monitor mitochondrial Ca^2+^ uptake independently of ER content, we first emptied intracellular Ca^2+^ stores with 1 μM thapsigargin, an inhibitor of the sarco/ER calcium ATPase, in Ca^2+^-free extracellular buffer. Subsequent Ca^2+^ addition triggers increases in cytosolic and mitochondrial Ca^2+^ that were monitored using cytosolic- and mitochondrial-targeted aequorin probes, respectively. No differences were detected in patients versus controls, with the [Ca^2+^] within mitochondria mirroring the fluctuations in cytosolic [Ca^2+^] (Figs 8C and D).

**Figure 9:**
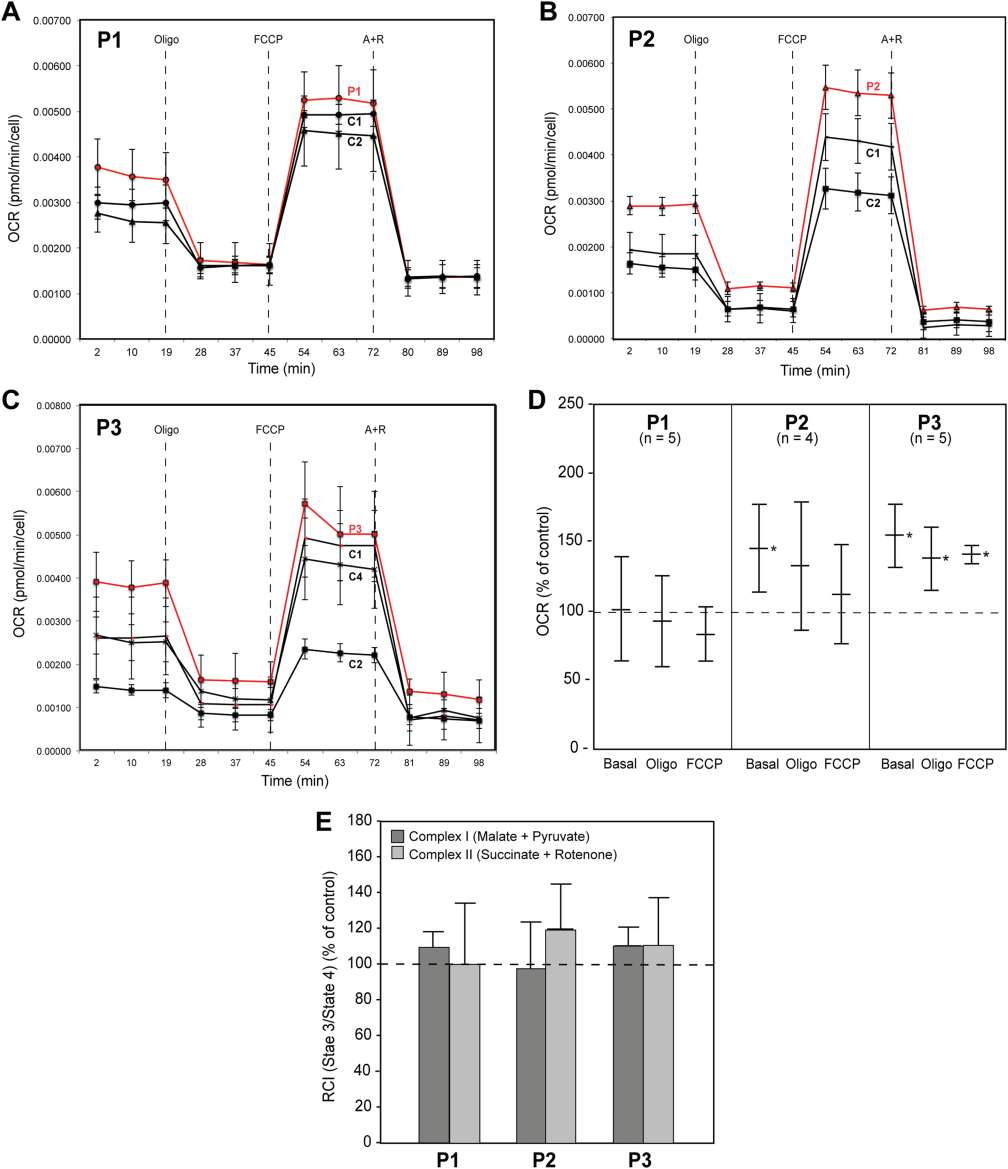
Respiratory chain activity in CMT2A^MFN2^ fibroblasts. **(A–C)** Representative Seahorse plots of OCR in the indicated patient and control whole cells using the Seahorse XF24 analyzer. Oligo, oligomycin; FCCP, carbonylcyanide p-triflouromethoxyphenylhydrazone; A + R, antimycin + rotenone. **(D)** Quantitation of OCR in whole patient cells relative to that in the control average (set at 100%). **(E)** RCI (state 3/state 4) via either complex I or complex II in permeabilized patient fibroblasts relative to the average of that in five controls. In all experiments *n* ≥ 3; ^*^P < 0.05.

MFN2 has been implicated in the control of capacitative calcium entry (CCE; the entry of Ca^2+^ from the extracellular medium upon depletion of intracellular stores) in cells with dysfunctional mitochondria (48). Whereas the cytosolic Ca^2+^ levels under resting conditions (‘R_min_’) were essentially unchanged (Fig. 8A), upon stimulation with thapsigargin CCE was increased significantly in patients 1 and 2 but not in patient 3 (Fig. 8B). Taken together, the data suggest that there are only moderate perturbations in calcium homeostasis in this disorder.

### Mitochondrial respiration in CMT2A^MFN2^ fibroblasts

MFN2 has been shown to play a key role in activating mitochondrial oxidative metabolism (49–51), which is also regulated by the connectivity between ER and mitochondria (30). Oscillations in the trafficking of calcium between ER and mitochondria via the MAM (29,40,41,52) are signals to induce aerobic metabolism (53), in part because Ca^+2^ within mitochondria tightly regulates the TCA cycle by activating matrix dehydrogenases in most (54), but not all (55), cells.

We therefore asked if mutations in CMT2A^MFN2^ fibroblasts affected bioenergetics. We measured the oxygen consumption rate (OCR) in patient and control cells using Seahorse technology (see representative individual experiments in Fig. 9A–C). There was a significant increase in baseline respiration and after the addition of specific respiratory complex inhibitors in patient 3, and in baseline OCR in patient 2, whereas the OCRs in patient 1 were similar to control values (Fig. 9D). Importantly, the changes in respiration were not due to an alteration in mitochondrial biogenesis (Fig. 2B), the synthesis of respiratory chain components (Supplementary Material, Fig. S6), or in mtDNA content (Fig. 2C). We also calculated the respiratory control index (RCI), a measure of the coupling of respiration to phosphorylation. To do that, we first measured baseline OCR in permeabilized cells via both complex I (i.e. in the presence of malate + pyruvate) and complex II (i.e. in the presence of succinate + rotenone) (both denoted state 2 respiration), followed by the sequential addition of ADP (state 3 respiration), oligomycin (state 4 respiration) and FCCP (uncoupled respiration) and calculated the ratio of state 3/state 4 respiration (i.e. RCI). There was no significant difference in either complex I- or complex II-mediated RCI between patients and controls (Fig. 9E).

We also measured mitochondrial membrane potential with TMRM. We found no difference in the uptake and retention of this membrane potential-responsive dye in patient versus control cells, both in basal conditions and in the presence of oligomycin+FCCP or antimycin+FCCP (Supplementary Material, Fig. S7).

Overall, bioenergetic function in these CMT2A^MFN2^ cells was not impaired, in agreement with results obtained in *Mfn2-*knockout cells (56), and in fact was slightly increased in two of the three patients, especially under basal conditions.

## Discussion

The mechanism(s) underlying the pathogenesis of CMT2A due to mutations in *MFN2* has been elusive. One question has been the role of mitochondria in the pathogenic process. In particular, besides alterations in mitochondrial morphology and distribution, some groups have found that mutations in *MFN2* cause a decrease (23,51,57), an increase (58,59) or no change at all (18,25), in bioenergetic function. Second, MFN2, either alone or in partnership with its paralog MFN1, plays at least two different roles in the cell, namely inter-mitochondrial fusion and ER–mitochondrial tethering at the MAM (13,14), rendering the deduction of the underlying mechanism(s) particularly difficult. Yet another confounding issue has been the use of various cellular and animal models to study the disease, often coupled with the use of knockout, gene silencing and gene overexpression paradigms. This is especially problematic when dealing with a dominantly inherited disorder in which both WT and mutant alleles are expressed, and whose equilibrium may be important in determining pathogenesis (5). Finally, little attention has been paid to the role of MAM-mediated ER–mitochondria interactions in CMT2A^MFN2^ disease pathogenesis.

We addressed these issues by focusing on mitochondria and MAM behavior in fibroblasts derived from CMT2A^MFN2^ patients. Although fibroblasts may not mimic phenotypes found in peripheral nerve, we have found that alterations in MAM in the central nervous system are also seen in MAM present in non-neuronal tissue, and in fibroblasts in particular (30,32).

Genetically, the analysis of mRNA transcription from the WT and mutant alleles indicated that there was no diminution of steady-state levels of the mutant transcripts. Nevertheless, total *MFN2* transcription was doubled (in patients 2 and 3), but protein levels remained unchanged. This finding implies that there may be feedback between the amount of protein and the amount of mRNA produced, especially from the mutant allele, and that even though there may be a higher dynamic turnover of mutant transcripts compared to WT, at steady state equal amounts of WT and mutant protein are produced. This speculation would be consistent with a dominant-negative effect of the mutant allele, as MFN2 functions as a dimer (both with itself and with MFN1). In addition, our observation that the mitochondrial network in patient cells appeared normal implies that the endogenous level of MFN1 homodimers (together with the WT MFN2 homodimers and WT-MFN2/MFN1 heterodimers) is sufficient to maintain the mitochondrial network (19).

Our phenotypic data showed that there was great variability among the three patients, mainly in measurements of ER–mitochondrial connectivity and MAM functionality (Table 2). With respect to physical connectivity, ER–mitochondrial co-localization as measured by confocal microscopy was increased over controls in patient 1, but not in patients 2 and 3. However, by EM, two patients (1 and 3) had less, not more, connectivity, and in all three patients the size distribution of those contacts was skewed toward greater inter-organellar distance. These ostensibly conflicting results can be reconciled if confocal microscopy—a relatively low-resolution technique (35)—of ER–mitochondrial contacts reflects inter-organellar contact *length* more than it does inter-organellar *distance*, and, in fact, we had observed this very phenomenon previously (30,32). This is particularly evident in patient 1, where we found the highest degree of co-localization by confocal microscopy and the highest ERMICC (which takes into account the length of the contact sites and the inter-organellar distance) by EM.

**Table 2:** Overall summary of data on CMT2A^MFN2^ patients

**Analysis**	**P1**	**P2**	**P3**	**Figure**
***Patient information***				
Disease presentation	Severe	Moderate	Mild
*MFN2* mutation	R364W	M376V	W740S	1
***Genetics***				
*MFN2* mRNA	=	+	+	2D
*MFN1* mRNA	=	=	=	S1
% mutant *MFN2* mRNA (vs 50%)	=	=	=	1C
MFN2 protein	=	=	=	2A
***Morphology***				
Mito length by CM	=	=	=	3B
Mito perimeter by EM	=	=	=	3C
Mito surface area by EM	=	=	=	3D
Mito–ER co-localization by CM	+	=	=	4A
MERC distance by EM	+	=	+	4B
MERC length by EM	+	=	=	S3A
ERMICC by EM	+	=	=	S3B
***MAM-related functions***				
PtdSer synthesis	=	=	−	5
PtdEtn synthesis	=	−	−	5
ACAT1 activity (CE synthesis)	+	±	=	6
LD formation	+	=	±	7
Resting cytosolic Ca^2+^	=	=	+	8A
Extracellular Ca^2+^ uptake (CCE)	+	+	=	8B
Cytosolic Ca^2+^ after stimulation	=	=	=	8C
Mitochondrial Ca^2+^ after stimulation	=	=	=	8D
Resting ER Ca^2+^ (D1ER)	=	=	=	8E
***Mitochondrial bioenergetics***				
Mitochondrial mass	=	=	=	2B
mtDNA content	=	=	=	2C
Basal respiration (OCR)	=	+	+	9D
RCI	=	=	=	9E
Mitochondrial membrane potential	=	=	=	S7

No change (=), increased (+), decreased (−), or higher but borderline significance (±) versus controls; CM, confocal microscopy; EM, electron microscopy.

While images of ER–mitochondrial connectivity are informative, they provide only a static snapshot of the two organelles and do not provide information regarding the highly dynamic nature of the connections between mitochondria and ER that can be modulated by local changes in metabolism (60,61) and by the ‘on–off’ kinetics of the contacts between the two organelles (30,32). This issue is particularly relevant in CMT2A^MFN2^, where WT and mutant MFN2 proteins coexist, but in a currently unclear relationship. In this regard, the greater inter-organellar distance described above was also reflected in the dynamic phospholipid synthesis experiments, most notably in the reduction in PtdEtn synthesis in patients 2 and 3. Interestingly, patient 1, with no reduction in PtdEtn synthesis, was the only one with consistent changes in ER–mitochondrial connectivity, suggesting that longer segments of contact might be able to compensate for the greater width separating the two organelles in this patient.

In contrast to the phospholipid data, there was an increase in MAM-mediated CE synthesis and LD formation, especially in patient 1. Thus, even in cells in which phospholipid transfer was unchanged, ACAT1 activity was increased (i.e. in patients 1 and 2), whereas in cells in which phospholipid transfer was decreased, ACAT1 activity was unchanged (i.e. in patient 3). We think that this curious result may actually provide insight into the mechanism by which MAM regulates its various functions (discussed further below).

A greater inter-organellar distance implies that functional biochemical crosstalk between ER and mitochondria ought to be reduced, including reduced ER–mitochondria Ca^2+^ exchange (41). Using a series of Ca^2+^ reporters targeted to different subcellular compartments, Ca^2+^ homeostasis did not appear to be altered, with the one exception of a significant increase in CCE [also known as store-operated calcium entry (SOCE)] (62) in patients 1 and 2. CCE occurs through so-called calcium release-activated channels (CRACs), which are mediated by the Ca^2+^ sensor STIM1, located at the plasma membrane, and the CRAC subunit ORAI1, located at the ER (63).

With respect to bioenergetics, here too there was variation among the three patients, similar to the variable results found by others (18,22,23,58): patients 2 and 3 showed increased basal respiration (OCR) versus controls, whereas patient 1 showed no changes. However, the RCI, which is a measure of the coupling between substrate oxidation and ATP production, showed no difference versus control in all three patients. Thus, in broad view, mitochondrial bioenergetics was undiminished in our patients. We also note that there was an inverse relationship between OCR and phospholipid transfer/synthesis in patients 2 and 3, implying that ER–mitochondrial contact plays a role in regulating bioenergetic output, as has been seen by others (30).

The mitochondrial abnormalities found by us, and by others (9,51,57), cannot be explained simply as a result of alterations similar to those seen in authentic mitochondrial disorders due to respiratory chain deficiency (64). Thus, in broad view, our data indicate that even though some aspects of bioenergetics may be perturbed in the disease, CMT2A^MFN2^ does not behave like an ‘authentic’ mitochondrial respiratory chain disorder (64,65).

Another major finding of this work is that CMT2A^MFN2^ cannot be pigeonholed into a single pathogenic entity, as there was phenotypic variability among the three patients. However, we reasoned that patient 1, with the most severe clinical phenotype, ought to be able to reveal most clearly what relationship, if any, exists between MAM behavior and pathogenesis. In fact, the data on patient 1 were contradictory. On the one hand, patient 1 had the most severe MAM-related phenotypes, namely, significant alterations in ER–mitochondrial contacts, CE synthesis, LD formation and extracellular calcium uptake. On the other hand, patient 1 showed no changes in other MAM-related phenotypes, including phospholipid transfer/synthesis and respiratory chain function. How can these two sets of data be reconciled?

We note that phospholipid synthesis and bioenergetics are fundamentally mitochondrial functions, whereas CE synthesis and LD formation are fundamentally ER functions. Thus, the two classes of functions could reflect two different aspects of MAM behavior, one ‘vertical’ (i.e. communication between the ER and the mitochondria) and the other ‘horizontal’ (i.e. functioning mainly within the MAM subdomain of the ER itself; 32). For example, phospholipid synthesis/trafficking is fundamentally ‘vertical’; it requires the intimate communication between both organelles, as the PtdSer that is made in the MAM subdomain of the ER is transferred to the mitochondrion, where it is converted to PtdEtn (37). On the other hand, CE synthesis is fundamentally ‘horizontal’; it requires the activity of ACAT1, which is located exclusively in the ER at the MAM subdomain, with the mitochondria playing essentially no metabolic role in the process (but likely modulating the lipid composition of the apposing MAM and MOM membranes; 30). Thus, a reduction in ER–mitochondrial communication in CMT2A^MFN2^ might disrupt ‘vertical’ processes more than it does ‘horizontal’ ones. This hypothesis is the subject of ongoing investigation.

**Figure 10:**
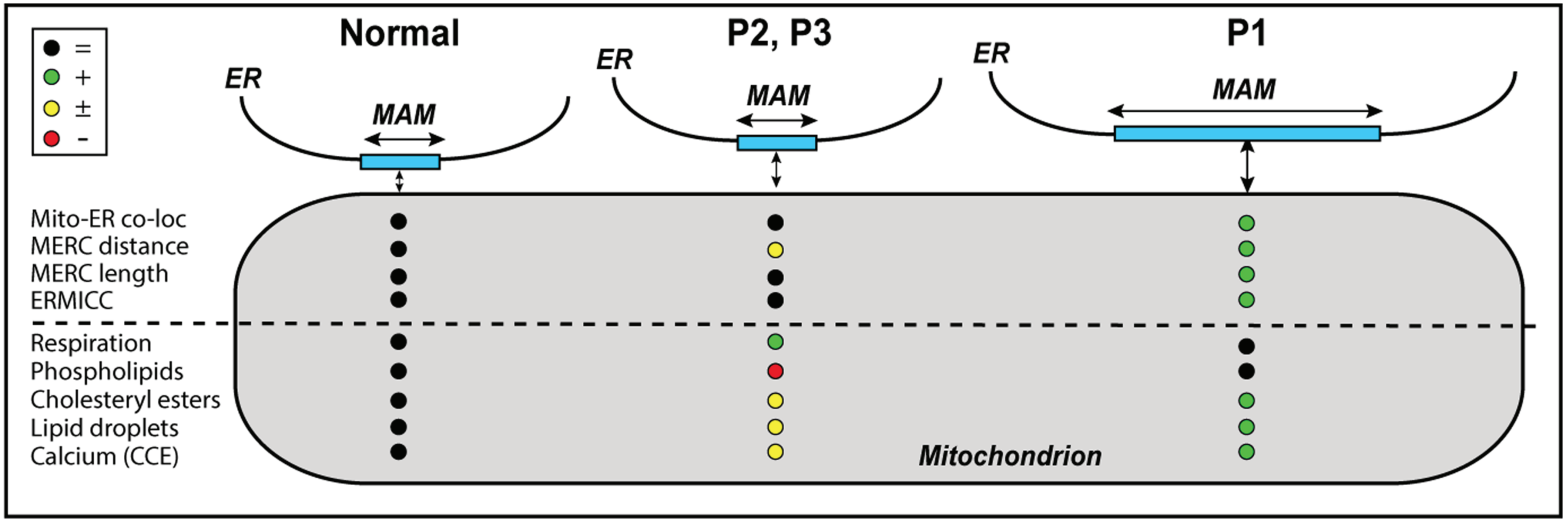
Working model of pathogenesis in CMT2A^MFN2^. The severity of the disease may be related to the degree of ER–mitochondrial communication. See text for details. Key at upper left (nomenclature as in Table 2).

The various phenotypes observed among the three patients have led us to develop a working model of pathogenesis consistent with the phenotypes seen in the CMT2A^MFN2^ cells analyzed here (Fig. 10). We note that ER–mitochondrial communication in Patient 1, with the severest phenotype, appears to differ from that in normal cells in two ways: greater inter-organellar *length* and greater inter-organellar *distance*, with the effects on MAM function of increased contact distance being partially compensated for by the increased contact length. This could explain why lipid metabolism and respiration—both ‘vertical’ functions—were essentially normal in this patient, whereas cholesterol metabolism and LD formation—both ‘horizontal’ functions—were increased. On the other hand, in Patients 2 and 3, with milder clinical phenotypes, contact distance was increased slightly whereas contact length was unchanged, which could help explain the reduced phospholipid metabolism, the moderate changes in cholesterol metabolism and the increased respiration. Whether these morphological differences are related to the location of the mutation within the MFN2 protein is currently unclear. Obviously, much more work needs to be done, especially in neuronal cultures, to determine the validity, and the limitations, of the model.

We believe that the altered MAM phenotypes that we observed in our fibroblasts can be extrapolated to other CMT-relevant cells, such as sensory neurons and Schwann cells. Because MAM regulates the synthesis of the main lipid components of myelin (30), impairment of the MAM ‘lipid factory’ could well explain why mutations in MFN2, a ubiquitously expressed protein, could be responsible for the loss of myelin found in CMT2A (9). In addition reduced levels of PtdEtn could also affect synaptic vesicle formation and membrane fluidity in peripheral neurons (66,67). Finally, in a peripheral neuropathy such as CMT2A^MFN2^, a possible pathogenetic mechanism is altered mitochondrial positioning and movement that likely affect the synaptic function and maintenance (9,25,27,28). The results reported here are not incompatible with such a view, as MAM (68) in general, and MFN2 in particular (69), are intimately associated with mitochondrial dynamics, including movement along microtubules.

Taken together, our findings could have implications for the treatment of this disorder and might even provide a conceptual basis underlying the pathogenesis of both axonal and demyelinating forms of the disease.

## Materials and Methods

### Patient cells and reagents

Patient 1 is a severely affected 32-year-old female with a R364W mutation in *MFN2*. She had normal early developmental milestones and began walking independently at 8 months of age. Her parents first noted mobility and balance difficulties at 2 years 7 months of age. She could never ride a bicycle. She has significant problems with fine motor activity of both hands and has been unable to cut food by herself since childhood. She requires a pedestal walker and long leg braces to ambulate. Her voice has been ‘hoarse’ since age 16, she has documented vocal cord paralysis and develops shortness of breath after speaking for several minutes. On neurological examination, she has weakness in biceps and triceps muscles in her upper extremities and no movements in her intrinsic hand muscles, wrist extensors or wrist flexors. In her lower extremities she has trace movements of her hamstrings and no movements of her quadriceps, anterior tibialis, gastrocnemius or intrinsic foot muscles. Small fiber sensory modalities, such as pin prick or light touch, are essentially normal, with only a mild decrease to pin prick on her right great toe. Large fiber sensory modalities are profoundly abnormal, as she was unable to detect vibration with a Rydell tuning fork at her toes, ankles, knees and fingers. Joint position was absent at her toes and left ankle, and reduced at both knees. Compound muscle action potential (CMAP) and sensory nerve action potential (SNAP) amplitudes were unobtainable except for a markedly reduced axillar nerve CMAP. Her CMT Neuropathy score was in the severe range of 28 out of 36 (70).

Patient 2 is a 62-year-old man with a mild clinical presentation of a M376V mutation in *MFN2* that has been described in detail (71). In brief, he developed progressive bilateral weakness of his feet and legs, which began at age 11. This was associated with gradual clawing of his feet, bilateral hand weakness, bilateral mild numbness of his hands and feet and foot pain. No bladder or bowel disturbance was reported. His sister, father and paternal grandmother also had a similar clinical presentation, suggesting autosomal dominant inheritance. On examination his cranial nerves were normal. He had a Medical Research Council (MRC) grade 4/5 weakness and wasting of the intrinsic muscles of the hand. He also had bilateral *pes cavus*, wasting of the calf muscles and MRC grade 4/5 weakness of hip and knee flexion and extension, and 3/5 weakness in the feet. Achilles tendon reflexes were absent but the remaining deep tendon reflexes were normal, with downgoing plantar responses. Mild sensory loss to pinprick was present below the elbows and knees. Vibration sensation was reduced below the ankles, proprioception was normal throughout and Romberg test was negative. No cerebellar dysfunction was detected. He had a high stepping gait.

Patient 3 is a 43-year-old female with a mild clinical phenotype and a W740S mutation in *MFN2*. Her early milestones were on time and she participated in all normal activities as a child, although she was a slow runner. She could ride a bicycle and ice skate, though not well because of balance. At her recent visit she noted decreased ability to feel touch from her toes to above her ankles and that she could no longer wear low-heeled shoes. She was not wearing ankle support although she felt unstable walking. She had no problems with fine motor function with her hands for activities, such as buttoning clothes, fastening jewelry or cutting food. She noted some difficulty in projecting her voice loudly. All muscles evaluated in both upper and lower extremities were graded as full strength (5/5) on neurological examination. Sensory examination revealed a mild reduction to pin prick and light touch sensation at her toes and decreased but present vibratory and position sense at her great toes. Her gait was judged to be normal except that she could not walk on her heels. Nerve conduction studies revealed reduced peroneal CMAP, absent sural and reduced median SNAP amplitudes, but were otherwise normal. CMT NS was 7 out of 36, in the mild range.

### Cell culture and analysis

Information on patient and control fibroblasts are summarized in Table 1; three of the controls were obtained from the Coriell Institute for Medical Research (Camden, NJ) and two were from the Columbia University Medical Center (CUMC). Cells were grown in Dulbecco's modified Eagle medium (DMEM) (Gibco Thermo Fisher Scientific, Rockford, IL; #11995–065) or minimum essential medium (MEM) (Gibco #11095–080), as indicated. For western blotting and immunocytochemistry, we used primary antibodies recognizing MFN1 (Abcam ab104274), MFN2 (Abcam, Cambridge, United Kingdom; ab50838), TOM20 (Santa Cruz, Dallas, TX; FL-145), α-tubulin (Sigma-Aldrich; T6199), protein disulfide isomerase (PDI; Cell Signaling Technology; #3501) and VDAC1 (Abcam ab15895). For western blot we used anti-rabbit IgG (Sigma-Aldrich; NA934V) or anti-mouse IgG (Sigma-Aldrich; NA931V) horseradish peroxidase-linked whole antibody as secondary antibodies. For immunocytochemestry, we used Alexa Fluor 488 goat anti-rabbit IgG (Invitrogen, Carlsbad, CA; A-11008) and Alexa Fluor 594 goat anti-mouse IgG (Invitrogen; A-11005).

Thin layer chromatography (TLC) silica plates were from EMD Biosciences (Millipore Sigma, Burlington, MA; 5748-7). PtdSer (#P7769), PtdEtn (#60648), cholesteryl palmitate (#C6072), cholesteryl oleate (#C9253) and lipid markers for TLC (#P3817) were from Sigma-Aldrich. Radiolabelled ^3^H-serine (NET248005MC) and ^3^H-cholesterol (NET139001MC) were from PerkinElmer (Waltham, MA); fatty acid-free bovine serum albumin (FAF-BSA) was from MP Biomedicals (Santa Ana, CA; #820472).

### Extraction of nucleic acids

Total DNA was extracted from fibroblast cultures by standard procedures (Puregene; Gentra Systems, Minneapolis, MN) according to the manufacturer’s instructions. Total RNA was extracted from fibroblasts using TRIzol Reagent (Invitrogen 15596–018) according to the manufacturer’s instructions. Both DNA and RNA were quantified with a NanoDrop2000 (Thermo Fisher Scientific).

### *MFN2* mutation verification

*MFN2* exons (see Fig. 1A) were amplified by PCR using primers (5′→3′): (RFLP-F-R364W-M376V: CACATGGAGCGTTGTACCAG and R-Seq R364W-M376M: CAGTAAGAGTCCAAGACTGCAGA; F-W740S: AGCGTCCTTAGGATGGTGCC and R-W740S: GCTTCATTCTCTTGGCAGTGGCC) and amplified DNA fragments were sequenced using the same forward and reverse primers. Thermal cycles for PCR amplification were 95°C for 5 min for initial denaturation; 35 cycles at 95°C for 30 s each; 55°C for annealing and 72°C for extension.

### cDNA synthesis

For RFLP analysis cDNA was synthesized from total RNA isolated from cells by a first strand cDNA synthesis kit using oligo-dT as a primer (Life Technologies Thermo Fisher Scientific) according to the manufacturer’s protocol. cDNA was amplified using primers RFLP-F-R364W-M376V: CACATGGAGCGTTGTACCAG; RFLP-R-R364W-M376V: TCGACCCAGTCCTTCCTCTA; hMFN2-1925F: CCCTCTCCTTTGGGCTCTAT; and hMFN2-2265R: CCTCAGGTGCCCACTATCTG, and sequenced with the same primers.

### Quantitative RT-PCR

We obtained cDNA using a High Capacity cDNA Reverse Transcription Kit (Applied Biosystems, Foster City, CA; 4368813, 4374966). Real-time PCR was performed in triplicate, with three technical replicates per experiment, in a StepOnePlus Real-Time PCR System (Applied Biosystems; #4376600). The expression of each gene under study was analyzed using specific *MFN2* (Hs00208382) and PGC-1α*PPARGC1A*; Hs01016719), normalized to *GAPDH* expression (Applied Biosystems, 4352339E) as an internal standard. Mitochondrial DNA content was measured as the ratio of mitochondrial DNA/nuclear DNA using the following set of primers and probes: mito_F: 5′-CCACGGGAAACAGCAGTGATT-3′; mito_R: 5′- CTATTGACTTGGGTTAATCGTGTGA-3′, Mito-probe: FAM-TGCCAGCCACCGCG-MGB (custom) and Nuclear-probe: TaqMan RNAase P control reagent kit (Thermo Fisher Scientific; #4316831).

### RFLP analysis

To determine the level of steady-state transcripts derived from the WT and mutant *MFN2* alleles, we performed RT-PCR on total RNA with appropriate primers and cleaved the resulting products with a restriction enzyme specific for either the WT or mutant alleles. For the R364W mutation, the C→T mutation at nt-1398 (numbering of GenBank accession #NM_001127660.1) results in loss of a *Hpa*II site. For the M376V mutation, the A→G mutation at nt-1434 results in loss of an *Nla*III site. Amplicons for both mutations were obtained using primers RFLP-F-R364W-M376V and RFLP-R-R364W-M376V. For the W740S mutation, the G→C mutation at nt-2527 neither creates nor destroys a restriction site; accordingly, we used a ‘mismatch’ forward PCR primer at this site that introduced an A at nt-2525 instead of a T [5′-GCAAAGCTGCTCAGGAATAAAGCCGG**A**T-3′; mismatch in bold (Supplementary Material, Fig. S2)] together with a reverse primer at the 3′ end of the transcript [5′- GCTTCATTCTCTTGGCAGTGGCC-3′], resulting in the gain of an *Mbo*I site in the mutant allele. Following polyacrylamide gel electrophoresis of the cleaved PCR products, we quantitated the intensity of the fragments by Image J and calculated the proportion of transcripts derived from each allele. Importantly, we normalized the intensity of the relevant cleaved fragment both for length (normalized to the length of its uncleaved precursor) and for the formation of uncleavable heteroduplexes (i.e. a ‘square root’ correction of the fraction of total signal derived from the WT allele, according to the Hardy–Weinberg law; 72).

### Mitochondrial respiration

Respirometry of cultured cells was performed using the XF24e Extracellular Flux Analyzer (Seahorse Bioscience; Agilent, Santa Clara, CA). We seeded an equal number of fibroblasts (60 000 cells) in each well. OCR was measured in basal conditions (Seahorse media with 2 mm pyruvate and 25 mm glucose) and after the addition, sequentially, of 1 μm oligomycin, 0.75 μm carbonylcyanide p-triflouromethoxyphenylhydrazone (FCCP) and 1 μm rotenone/1 μm antimycin A. All OCR values were normalized to cell number after the experiment. All results were averages of at least three technical replicates of more than four biological replicates.

For permeabilization assays (60 000 cells/well), the culture medium was replaced by the mitochondrial assay solution (220 mm mannitol, 70 mm sucrose, 5 mm KH_2_PO_4_, 5 mm MgCl_2_, 2 mm HEPES, 1 mm EGTA and 0.2% FAF-BSA, pH 7.4) containing 10 nm of the XF Plasma membrane permeabilizer reagent XF PMP (Seahorse Bioscience #102504–100) and 5 mm malate + 5 mm pyruvate (for complex I assays) or 5 mm succinate + 2 μm rotenone (for complex II assays). OCR was measured first with no added substrates (state 2), and then after the sequential addition of 3 mm ADP (state 3), 4 μm oligomycin (state 4) and 6 μm FCCP (uncoupled respiration).

### Measurement of mitochondrial membrane potential

For analysis of mitochondrial transmembrane potential, 2000 fibroblasts per well of the indicated genotype were plated onto 384 well dishes (CellCarrier, PerkinElmer). After 24 h in culture, cells were loaded with 10 nm of the potentiometric dye tetramethylrhodamine methylester (TMRM), as described previously (73,74). Briefly, cells were rinsed in 10 mm HEPES buffered saline (HBSS buffer, pH 7.4; Invitrogen) and subsequently loaded with 10 nm TMRM in the presence of 1 μm cyclosporine H (30 min at 37°C) and left in the same buffer during image acquisition. Alternate brightfield and fluorescence (excitation/emission at 520–550/560–630 nm) images were acquired every 3 min, using the 20× magnification air objective of the high content screening (HCS) imaging system Operetta® and Harmony® software (PerkinElmer). Where indicated, cells were challenged with either 1 μm oligomycin or 2 μm antimycin A; the ionophore FCCP (2 μm) was added as a control for mitochondrial depolarization. For image analysis, clusters of mitochondria were identified as regions of interest and the average fluorescence intensities after background subtraction were obtained using the Image J (NIH) software and normalized for comparative purposes.

### Analysis of phospholipid synthesis in cultured cells

Cells were incubated for 2 h with serum-free medium to ensure removal of exogenous lipids. The medium was then replaced with MEM containing 2.5 μCi/ml of ^3^H-serine for the indicated periods of time. The cells were washed and collected in DPBS, pelleted at 2500 g for 5 min at 4°C and resuspended in 0.5 ml water, removing a small aliquot for protein quantification. Lipid extraction was done by the Bligh and Dyer method (75). Briefly, three volumes of chloroform/methanol 2:1 were added to the samples and vortexed. After centrifugation at 8000 g for 5 min, the organic phase was washed twice with two volumes of methanol/water 1:1, and the organic phase was blown to dryness under nitrogen. Dried lipids were resuspended in 60 μl of chloroform/methanol 2:1 (v/v) and applied to a TLC plate. Phospholipids were separated using two solvents, composed of petroleum ether/diethyl ether/acetic acid 84:15:1 (v/v/v) and chloroform/methanol/acetic acid/water 60:50:1:4 (v/v/v/v). Development was performed by exposure of the plate to iodine vapor. The spots corresponding to the relevant phospholipids (identified using co-migrating standards) were scraped and counted in a scintillation counter (Packard Tri-Carb 2900TR).

### Assay of ACAT activity

To measure ACAT activity *in vivo*, whole cells were incubated in serum-free medium for 2 h to remove all exogenous lipids. After that, 2 μCi/ml of ^3^H-cholesterol was added to fetal bovine serum (FBS)-free DMEM containing 2% FAF-BSA, allowed to equilibrate for at least 30 min at 37°C, and the radiolabelled medium was added to the cells for the indicated periods of time. Cells were then washed and collected in DPBS, removing a small aliquot for protein quantification. Lipids were extracted as described above and samples were analyzed by TLC along with an unlabeled CE standard. A mixture of chloroform/methanol/acetic acid 190:9:1 (v/v/v) was used as solvent. Iodine stains corresponding to CE bands were scraped and counted.

### LD staining

Staining of LDs was performed using HCS LipidTox Deep Green neutral lipid stain (Invitrogen H34475) according to manufacturer’s instructions. LD staining was quantified using ImageJ. When reported as intensities, the values in the text represent the product of the intensity and the area covered by the fluorescent signal above background divided total cell number. For each cell type, we took between 15 and 20 images for analysis (at least 5 images per experiment). The images were first taken at lower magnification (20×) in order to view 50–100 cells per field. Subsequently, we acquired the images at 63× for quantification. The numbers reported in the text represent the averages derived from the quantification of patients versus controls.

### Calcium measurements

For CCE measurements, cells were loaded with 2 μm Fura-2-AM (Thermo Fisher Scientific) in Krebs-Ringer modified buffer (KRB: 125 mm NaCl, 5 mm KCl, 1 mm Na_3_PO_4_, 1 mm MgSO_4_, 5.5 mm glucose, 20 mm HEPES, pH 7.4) supplemented with 1 mm CaCl_2_, 250 μm sulfinpyrazone and 0.02% pluronic acid for 30 min at 37°C. Cytosolic Ca^2+^ levels were measured in the absence of stimuli. Cells were then treated with 1 μm thapsigargin in KRB supplemented with 500 μm EGTA for 5 min at 37°C, in order to induce depletion of intracellular Ca^2+^ stores. Subsequently CCE was measured upon addition of 1 mm Ca^2+^. Both basal cytoplasmic and CCE values are reported as the fluorescence intensity ratios at 340/380 nm.

Intracellular and mitochondrial Ca^2+^ were measured using the calcium reporter aequorin. Cells were seeded in 96 well plates (10 000 cells per well). After 24 h, cells were infected with adenoviral particles encoding for cytosolic or mitochondrial-targeted aequorin-based Ca^2+^ probes (Italian Ministry of Health authorization number PD/1C/IMP2/10–001-08/03/10). Thirty-six h after infection, cells were incubated with 5 μm of the aequorin cofactor coelenterazine for 1 h in KRB at 37°C, supplemented with 1 mm CaCl_2_. Cells were then washed, treated with 1 μm thapsigargin in KRB supplemented with 100 μm EGTA. Luminescence from each well was measured for 50 s in an EnVision microplate reader (PerkinElmer) equipped with a two-injector unit. After 3 s from the beginning of the experiment, 100 μm Ca^2+^ was first injected to elicit an elevation in [Ca^2+^], and then a hypotonic, Ca^2+^-rich, 100 μM digitonin-containing solution was added to discharge the remaining aequorin pool. The light signal was collected, calibrated and converted to [Ca^2+^] values by an algorithm based on the Ca^2+^ response curve of aequorin at physiological conditions of pH, [Mg^2+^] and ionic strength, as described (76). Output data were analyzed and calibrated with a custom-made macro-enabled Excel workbook. All of the results are the average of three independent experiments expressed as mean ± SD.

To measure ER calcium, cells were plated at 3000 cells/well, with 90 μl of complete medium, in a 384 well plate (Cell Carrier, PerkinElmer). Twenty-four h after plating, cells were transfected with a mixture including 0.1 μg of plasmid D1ER, a Forster resonance energy transfer (FRET)-based Ca^2+^ probe targeted to the ER (47), 3 μl of GenJet (SignaGen Laboratories, Rockville, MD) and 10 μl of FBS-free DMEM. One day later, images were acquired by means of the Operetta High Content Imaging System (PerkinElmer). Image analysis was performed using Harmony 4.1 Image Analysis Software (PerkinElmer). FRET ratio was calculated as (FRET_cell_ − FRET_background_) / (CFP_cell_ − CFP_background_), where the FRET corresponds to the intensity of FRET channel, and CFP is the intensity of the donor cyan fluorescent protein (47). The two emission wavelengths were collected with 460–500 nm and 520–560 nm BandPass filters, respectively. Excitation was achieved with a Xenon lamp and a 410–430 nm BandPass filter.

### Analysis of mitochondrial morphology

Mitochondria were identified by immunocytochemistry using anti-TOM20, and their lengths were measured using Image J/FIJI (77). Images were taken using a SP8 confocal microscope (Leica). Mitochondrial perimeter was measured on EM images, using MitoLoc together with ImageJ software, as described (78). Experiments were performed on control and patient samples analyzed side-by-side, run on 3 different days (i.e. *n* = 3), with 5 technical replicates per sample.

### Analysis of ER–mitochondrial apposition by confocal microscopy

Cells were fixed in 4% paraformaldehyde and immunostained with a rabbit antibody to TOM20 to detect mitochondria and with a mouse antibody to PDI to detect ER. Secondary antibodies were goat anti-rabbit coupled to Alexa 594 and goat anti-mouse coupled to Alexa 488. Interactions between mitochondria and ER were calculated using Image J software (79), determining the area occupied by one organelle and using its signal as a mask for the other one, as described (80). The various co-localization data sets were compared using Mander’s coefficient. Co-localizations were performed on control and patient samples analyzed side-by-side, run on 3 different days (i.e. *n* = 3), with 5 technical replicates per sample.

### Transmission EM

Cells were cultured in 24 well plates. At 70–80% confluency, cells were washed once with PBS and fixed with pre-heated Fixative Buffer (0.1 M sodium cacodylate pH 7.4, 2.5% glutaraldehyde) for 30 min at room temperature. Cells were stored at 4°C for 90 min before replacing the medium with Storage Buffer (0.1 M sodium cacodylate pH 7.4). Thin slices of cells obtained after resin infiltration and polymerization were imaged on a Tecnai-20 electron microscope (Philips-FEI). Three sets of independent samples were prepared, and each sample was designated with a numerical code. Images from 6 different cells (total of 70 images) per sample were collected blinded. Mitochondrial–ER distance measurements were also carried out blind, using ImageJ software, setting 150 nm as the maximum distance, as described previously (14). This type of ‘static’ image analysis was validated by means of a FRET probe in which the acceptor and donor fluorophores were located at the surface of either organelle in live cells; hence, reduced ‘vertical’ distance between the two corresponds to a decreased FRET ratio (14). The advantage of using TEM analysis to calculate ERMICC gives us the possibility of including ER–mitochondrial distance, the length of the contacts and the mitochondrial perimeter in a single value (14,36).

## Supplementary Material

Supplementary Data
