# Supplementary material for: MFN2 mutations in Charcot–Marie–Tooth disease alter mitochondria-associated ER membrane function but do not impair bioenergetics
Source: Hum Mol Genet. 2019 Jan 11;28(11):1782–800. doi: 10.1093/hmg/ddz008 (PMC6522073; doi:10.1093/hmg/ddz008)
Supplement: Supplementary Data [file hmg-2018-twb-00882_supplemental_data_ddz008.pdf]

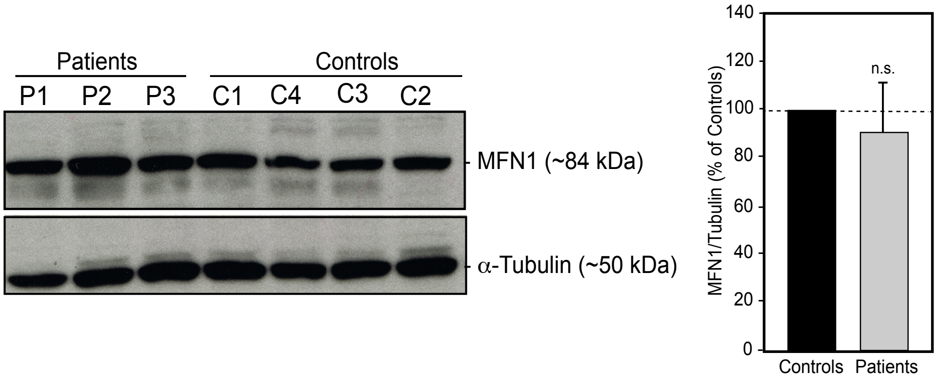

Supplementary Figure S1. Analysis of MFN1 expression in CMT2A^{MFN2} patients. Western blots to detect MFN1 protein in the indicated patients and controls relative to the expression of α-tubulin. 20 μg loaded in each lane. Quantitation at right. n = 3; n.s. not significant.

Fig. S2

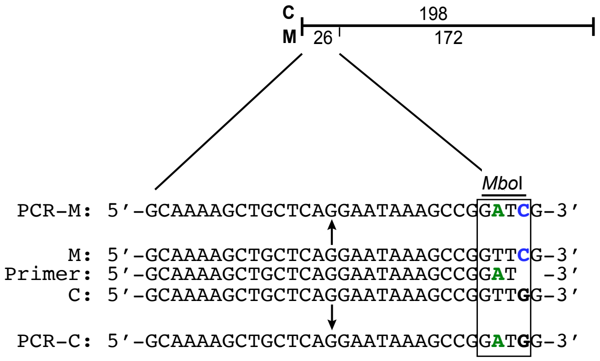

Supplementary Figure S2. Mismatched RT-PCR/RFLP strategy for W740S. Shown are the sequences of the control (C) and mutant W740S (M) alleles relative to that of the mismatched primer. Note that the mutant, but not the control, creates a novel *Mbo*I site (GATC). Map of the amplified region, and of the predicted cleaved fragments (in bp), at top.

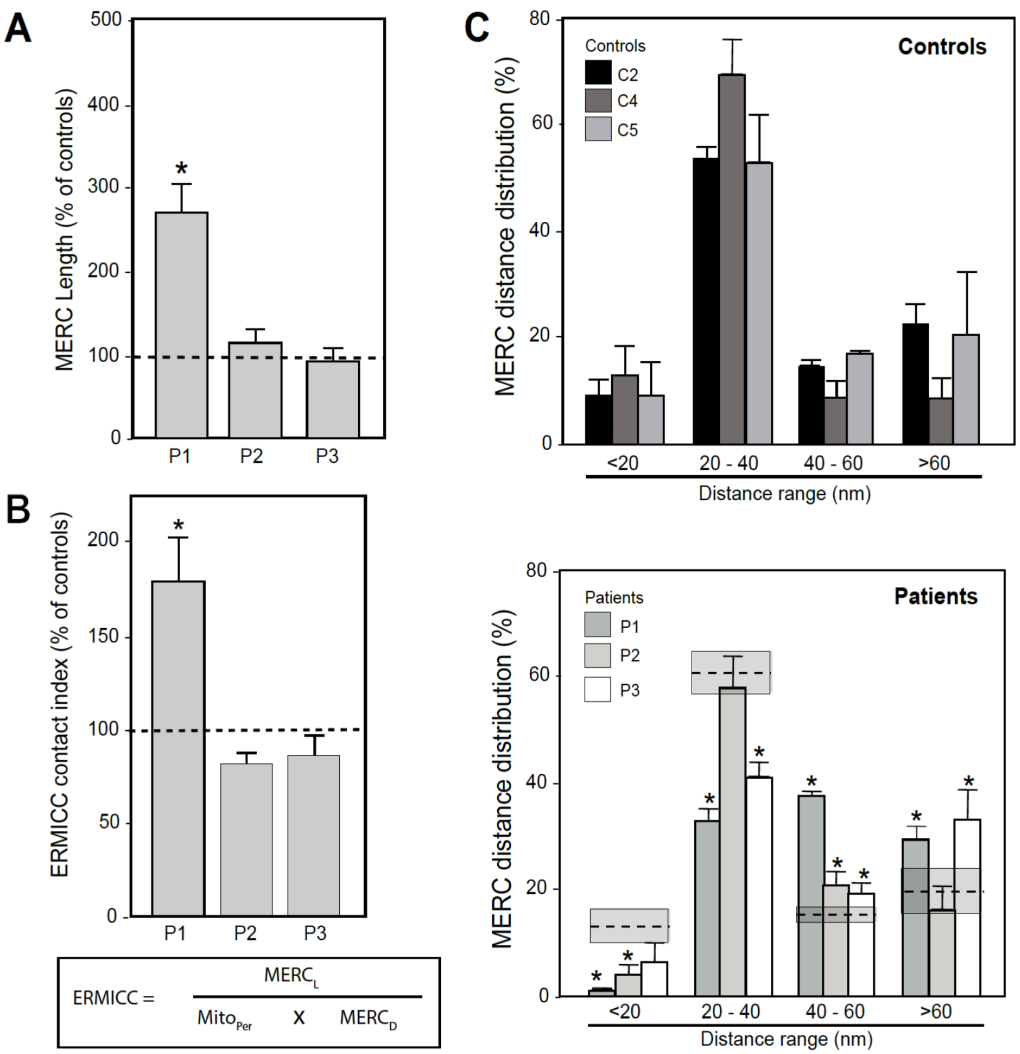

Supplementary Figure 3. Analysis of ER-mitochondrial connectivity. (A) MERC length (length of contact between ER and mitochondria, measured in nm) by EM. **(B)** ERMICC, which takes into account the ER-mitochondrial distance (D; see Fig. 4B), the mitochondrial perimeter (Per), and the length of the contacts (L); see formula [boxed]), in nm^{-1}, by EM. In both panels, the controls were set to 100% and the value in the patients' cells was normalized to that of the controls (n = 3). *, p<0.05. **(C)** Histograms of the distribution of ER-mitochondrial distances in the indicated controls (top) and patients (bottom). Each dotted line within a shaded box denotes the average ± SD of the control data in each size range shown in the upper panel. n = 3; *, p<0.05 in the patients vs average of the controls (shaded boxes) in each size range derived from the data shown in the upper panel.

Fig. S4

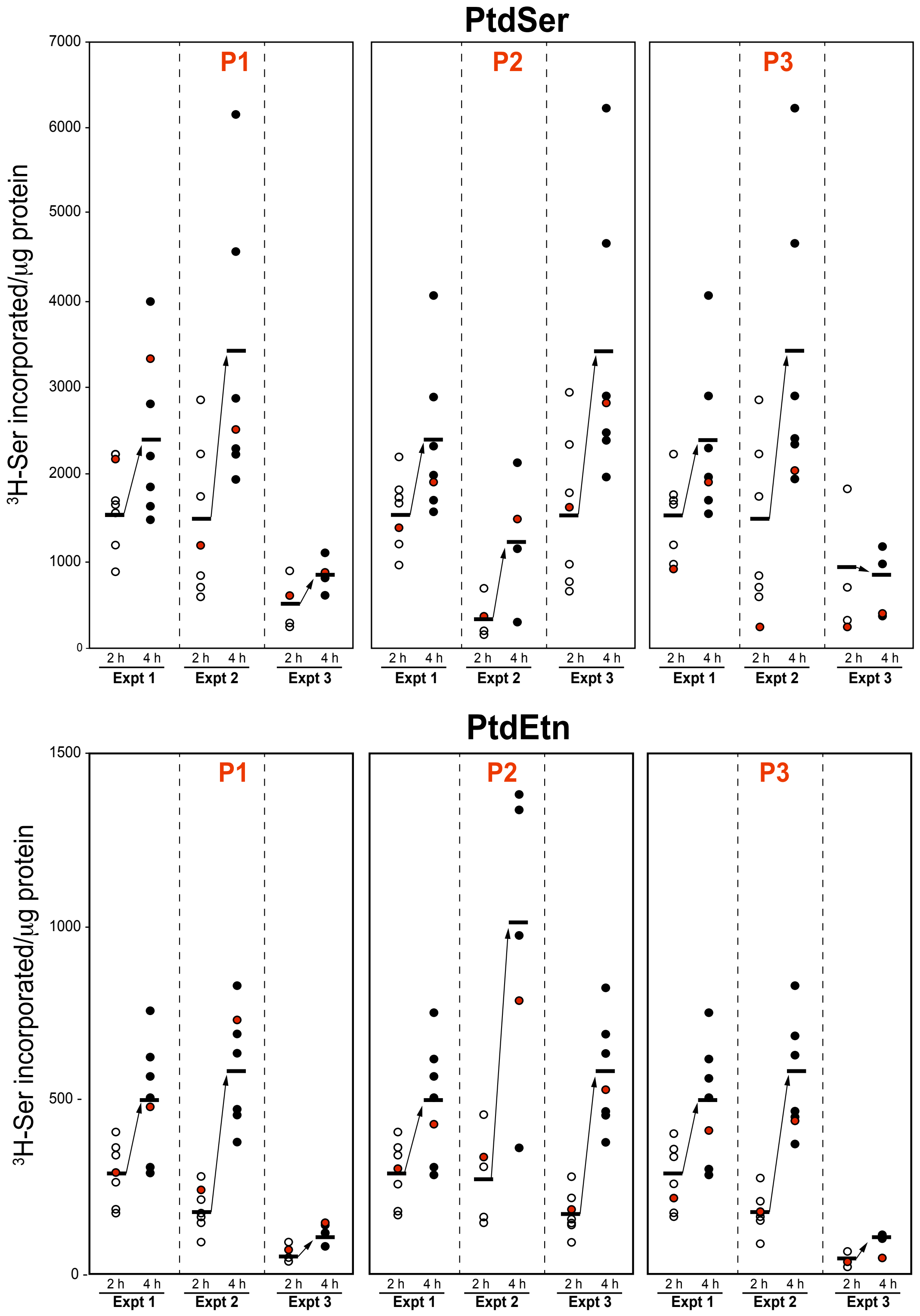

Supplementary Fig. S4. Phospholipid transport and synthesis in CMT2A^{MFN2} fibroblasts. Incorporation of ^{3}H-Ser into ^{3}H-PtdSer and ^{3}H-PtdEtn in the controls (black circles) after 2 h and 4 h, and in the patient cells (red circles). Bars denote average values of the controls. Note general increase in average ^{3}H-Ser incorporation between 2 h and 4 h (arrows), as expected.

Fig. S5

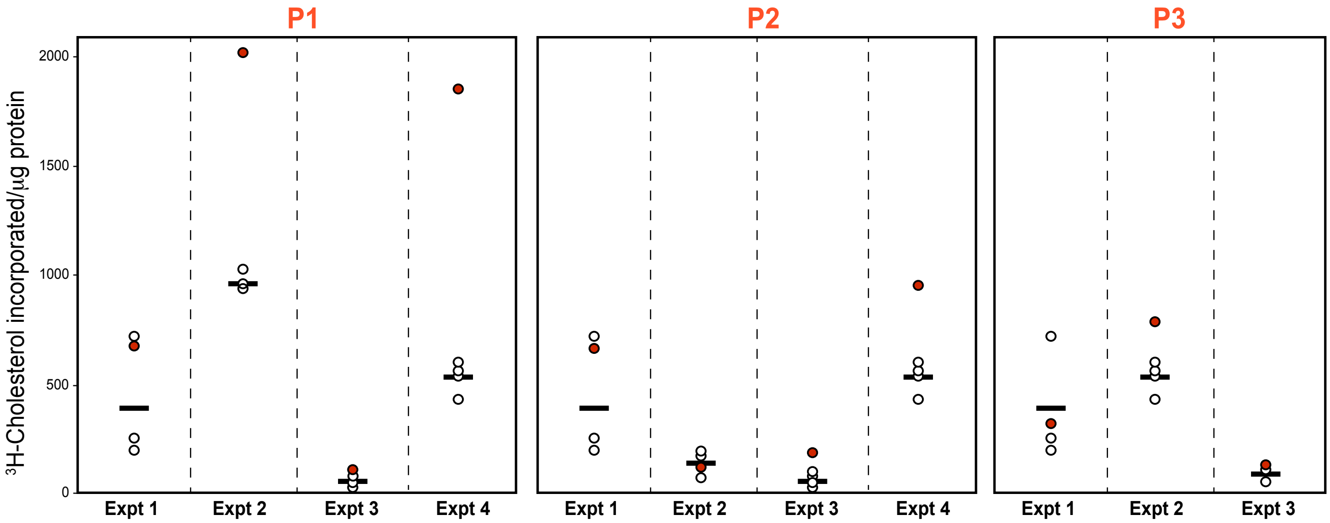

Supplementary Figure S5. Cholesteryl ester synthesis in patient fibroblasts. Incorporation of ^{3}H-cholesterol into ^{3}H-CE (i.e. ACAT1 activity) in the indicated patients (red circles) relative to controls (open circles) in separate experiments. Bars denote average of control values.

Fig. S6

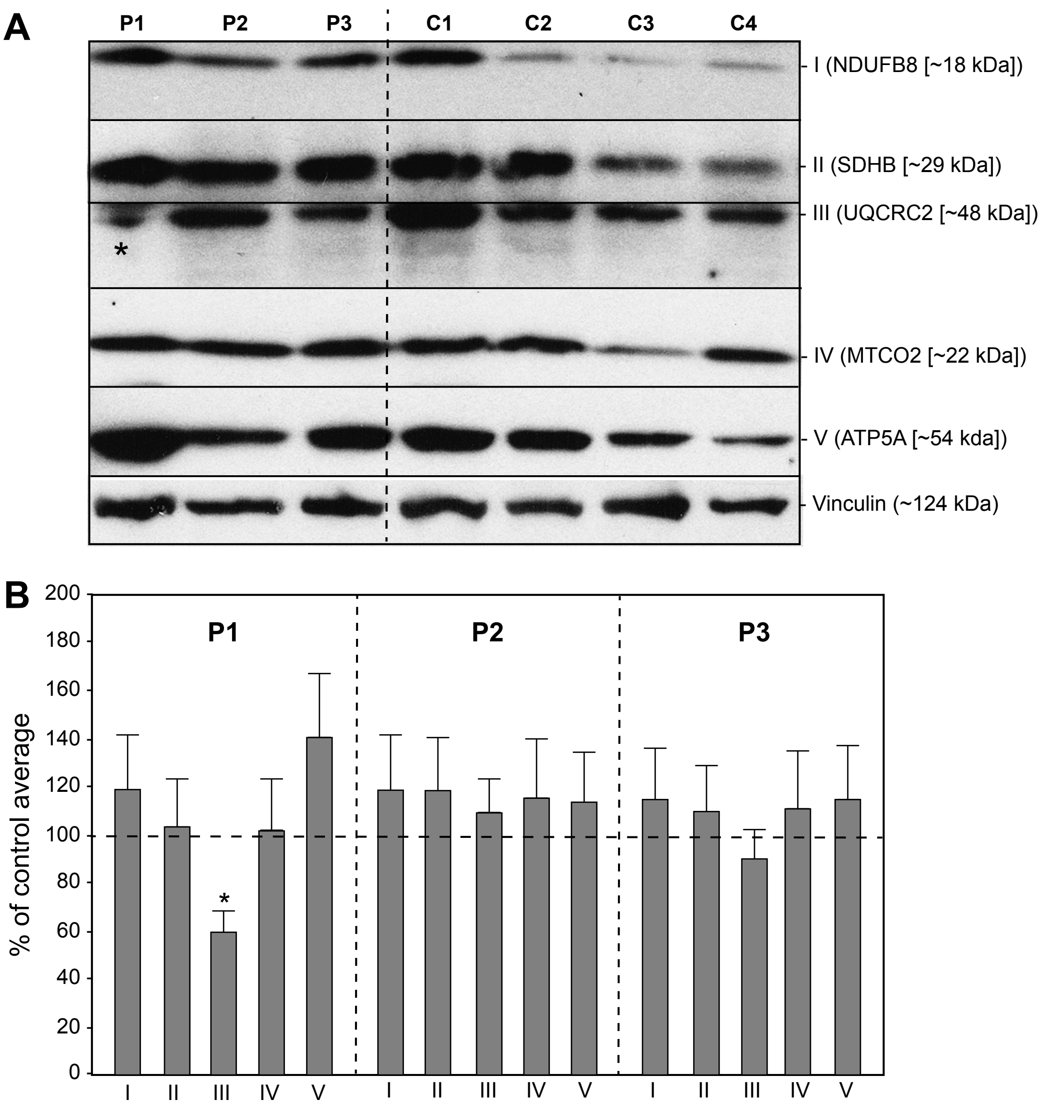

Supplementary Figure S6. (A) Western blot of representative subunits from respiratory complexes I–V, relative to that of vinculin. **(B)** Quantitation of the western signals in the three patients normalized to the average of the controls (n=4). The asterisk denotes an artifactually low signal in P1 due to a migration anomaly in the blot in panel A.

Fig. S7

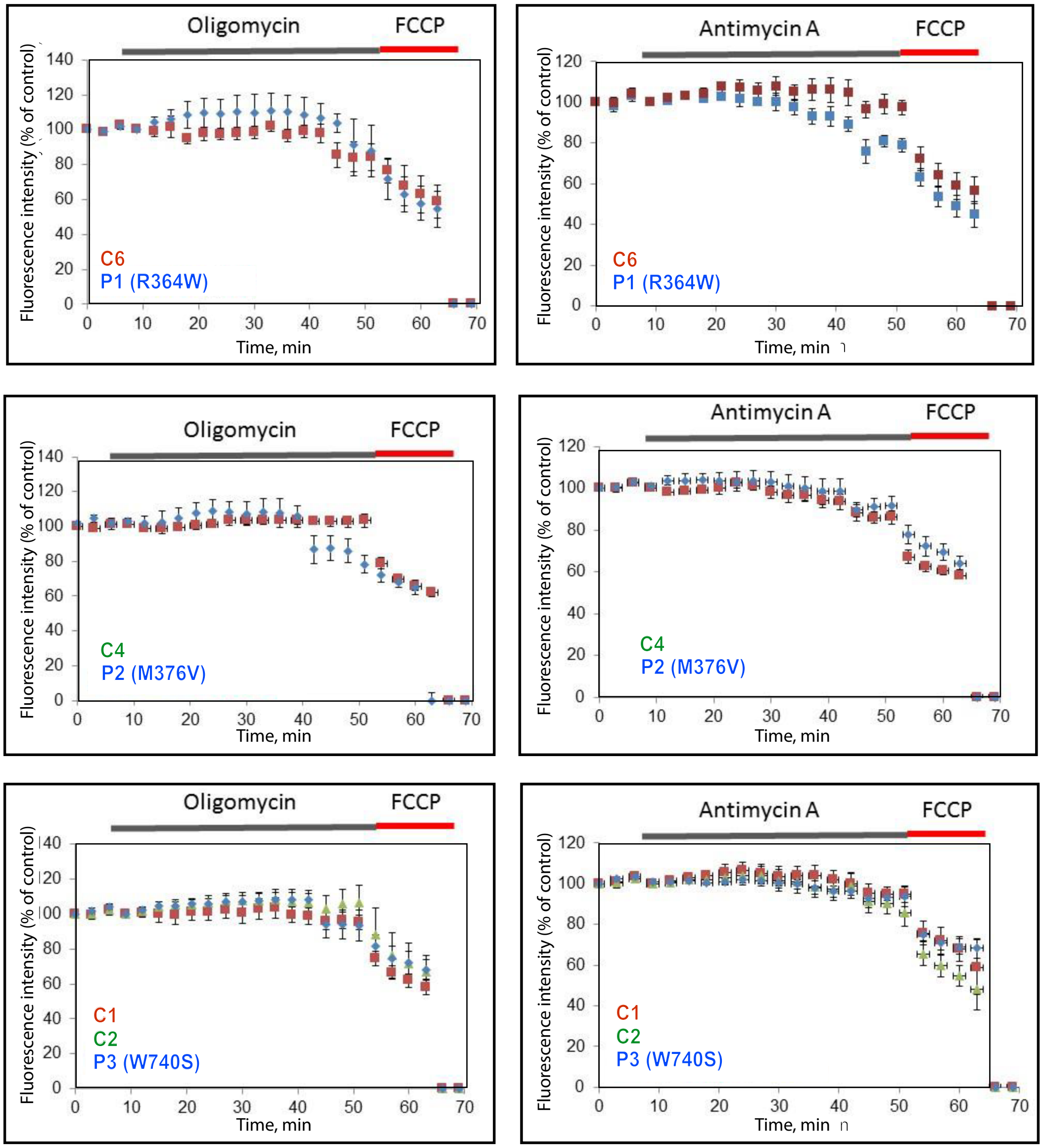

Supplementary Figure S7. Assessment of mitochondrial membrane potential. Measurement of fluorescence intensity in the presence of the membrane potential-responsive dye TMRM after treatment of the indicated cells with 1 μM oligomycin or 2 μM antimycin and 2 μM FCCP; $n \geq 4$.
